# Menin maintains lysosomal and mitochondrial homeostasis through epigenetic mechanisms in lung cancer

**DOI:** 10.1038/s41419-025-07489-0

**Published:** 2025-03-08

**Authors:** Jun-Bo Yuan, Gui-Xin Gu, Bang-Ming Jin, Qing Han, Bing-Hui Li, Li Zhang, Bin Xu, Xuan Zhu, Guang-Hui Jin

**Affiliations:** 1 Department of Basic Medical Sciences, School of Medicine, Xiamen University, Xiamen, Fujian PR China; 2 Fujian Provincial Key Laboratory of Innovative Drug Target Research, School of Pharmaceutical Sciences, Xiamen University, Xiamen, Fujian PR China; 3 State Key Laboratory of Cellular Stress Biology, Xiamen University, Xiamen, Fujian PR China

**Keywords:** Lung cancer, Organelles, Epigenetics

## Abstract

Lysosome-mediated autophagy (including mitophagy) is crucial for cell survival and homeostasis. Although the mechanisms of lysosome activation during stress are well recognized, the epigenetic regulation of lysosomal gene expression remains largely unexplored. Menin, encoded by the *MEN1* gene, is a chromatin-related protein that is widely involved in gene transcription via histone modifications. Here, we report that menin regulates the transcription of specific lysosomal genes, such as *CTSB*, *CTSE*, and *TFE3*, through MLL-mediated H3K4me3 reprogramming, which is necessary for maintaining lysosomal homeostasis. Menin also directly controls the expression of *SQSTM1* and *MAP1LC3B* to maintain autophagic flux in a manner independent of AMPK/mTORC1 pathways. Furthermore, loss of menin led to mitochondrial dysfunction, elevated levels of reactive oxygen species (ROS), and genome instability. In genetically engineered mouse models, *Men1* deficiency resulted in severe lysosomal and mitochondrial dysfunction and an impaired self-clearance ability, which further led to metabolite accumulation. SP2509, a histone demethylase inhibitor, effectively reversed the downregulation of lysosomal and mitochondrial genes caused by loss of *Men1*. Our study confirms the previously unrecognized biological and mechanistic importance of menin-mediated H3K4me3 in maintaining organelle homeostasis.

## Introduction

Epigenetic modifications, including histone modifications, play a crucial role in linking environmental cues, such as changes in metabolite levels, to the regulation of gene expression to maintain homeostasis [1]. Menin, encoded by the multiple endocrine neoplasia type 1 (*MEN1*) gene, acts as a necessary scaffold protein that interacts with writer proteins and participates in various histone modifications to regulate gene transcription and cellular phenotypes [2–5]. Mixed-Lineage Leukemia (MLL) is a member of the histone-lysine N-methyltransferase 2 (KMT2) family that specifically participates in histone H3 lysine 4 trimethylation (H3K4me3) to promote genome accessibility and transcription [6]. *MLL1* (*KMT2A*) and *MLL4* (*KMT2B*) share similar structural domains that interact with menin through its N-terminal domain [6]. Menin recruits MLL1 and MLL4 to specific chromatin sites and promotes the transcription of genes through H3K4me3 [5–7]. Menin also represses the expression of proliferation-promoting genes through protein arginine methyltransferase 5 (PRMT5)-mediated histone 4 arginine 3 di-methylation (H4R3me2) and suppressor of variegation 3–9 homolog 1 (SUV39H1)-mediated H3K9me3 in endocrine tumors [2, 3]. Menin can also act as a reader that specifically recognizes H3K79me2 marks on chromatin [4]. Due to its complex epigenetic characteristics, menin has diverse and tissue-specific biological functions. For example, *Men1* loss results in pancreatic islet tumor formation by inactivating the tumor suppressors cyclin-dependent kinase inhibitor-1c (*p27*^*Kip1*^) and -2c (*p18*^*INK4c*^) [5]. We reported that the interplay between KRAS (Kirsten rat sarcoma viral oncogene homolog) and menin plays an important role in regulating the development of lung cancer and that the loss of menin leads to neuroendocrine (NE) differentiation in KRAS mutant-induced lung adenocarcinoma [8, 9]. In contrast, menin is required for MLL-AFs fusion protein-mediated activation of homeobox (*HOX*) gene expression and leukemogenesis [7]. The intricate biological role of menin in controlling malignant transformation of cells needs further clarification.

Lysosomes maintain organelle homeostasis and protein integrity in eukaryotes. Lysosomes regulate metabolic signaling pathways, such as those related to nutrition and energy, and are crucial for macroautophagy/autophagy [10]. Autophagy-related proteins (ATGs) and microtubule-associated protein light chain 3 (LC3) proteins coordinate the formation of autophagosomes to recognize and engulf substrates labelled with selective autophagy receptors such as P62; ultimately, autophagosomes fuse with lysosomes to form autolysosomes, completing the degradation of the substrates [11]. Dysfunction of lysosomes and autophagy leads to various diseases, including cancer [10, 11]. Currently, extensive research has revealed the molecular mechanisms by which stress conditions regulate lysosomes and autophagy. Lysosomal genes are regulated primarily by transcription factors of the microphthalmia/transcription factor E (MiT/TFE) family, particularly TFEB and TFE3 [10]. Stressors, such as deprivation of amino acids, glucose, and growth factors, activate TFEs to form homo- or hetero-dimers [10]. These dimers bind to coordinated lysosomal expression and regulation (CLEAR) elements in the promoters of lysosomal and autophagic genes, including cathepsin B (*CTSB*), lysosomal-associated membrane protein 1 (*LAMP1*), *ATG5*, and *SQSTM1*, directly regulating their transcription [10]. Mechanistic target of rapamycin complex 1 (mTORC1) and 5’ adenosine monophosphate-activated protein kinase (AMPK) signaling indirectly regulate lysosomal gene expression through phosphorylation of TFEs, thereby modulating the nuclear-cytoplasmic shuttling of these TFEs [10]. One of the important functions of lysosomes is clearing damaged mitochondria, a process called mitophagy [11]. Mitochondria are critical organelles that provide cellular energy through oxidative phosphorylation [12]. A recent study found that lysosomal and mitochondrial biogenesis is simultaneously regulated by the AMPK-TFEB axis [13], suggesting a common mechanism for the regulation of lysosomes and mitochondria.

At present, the epigenetic mechanism of lysosome biogenesis has not been fully identified. Euchromatic histone lysine methyltransferase 2 (EHMT2) inhibits the transcription of autophagy genes, such as microtubule-associated protein 1 light chain 3 beta (*MAP1LC3B*, encoding the LC3B protein) and WD repeat domain phosphoinositide-interacting protein 1 (*WIPI1*), through H3K9me2 [14]. Coactivator-associated arginine methyltransferase 1 (CARM1) promotes the transcription of *MAP1LC3B*, *ATG14*, ATPase H+ transporting v1 subunit c1 (*ATP6V1C1*) and hexosaminidase subunit beta (*HEXB*) through histone 3 arginine 17 di-methylation (H3R17me2) [15]. Lysine-specific demethylase 1 (LSD1) is a histone H3K4 demethylase that removes H3K4me3 from chromatin through an unknown mechanism [16]. It has been reported that feeding increases the recruitment of LSD1 to the promoters of *TFEB* and *ATG3*, leading to transcriptional silencing through erasure of H3K4me2/3 [17]. The epigenetic mechanisms underlying the transcriptional regulation of lysosomal and mitochondrial genes need to be further elucidated. Here, we report that menin-MLL mediated H3K4me3 remodeling is a necessary epigenetic event for maintaining lysosomal and mitochondrial homeostasis.

## Results

### Menin maintains lysosomal enzyme expression and function

**Fig. 1:**
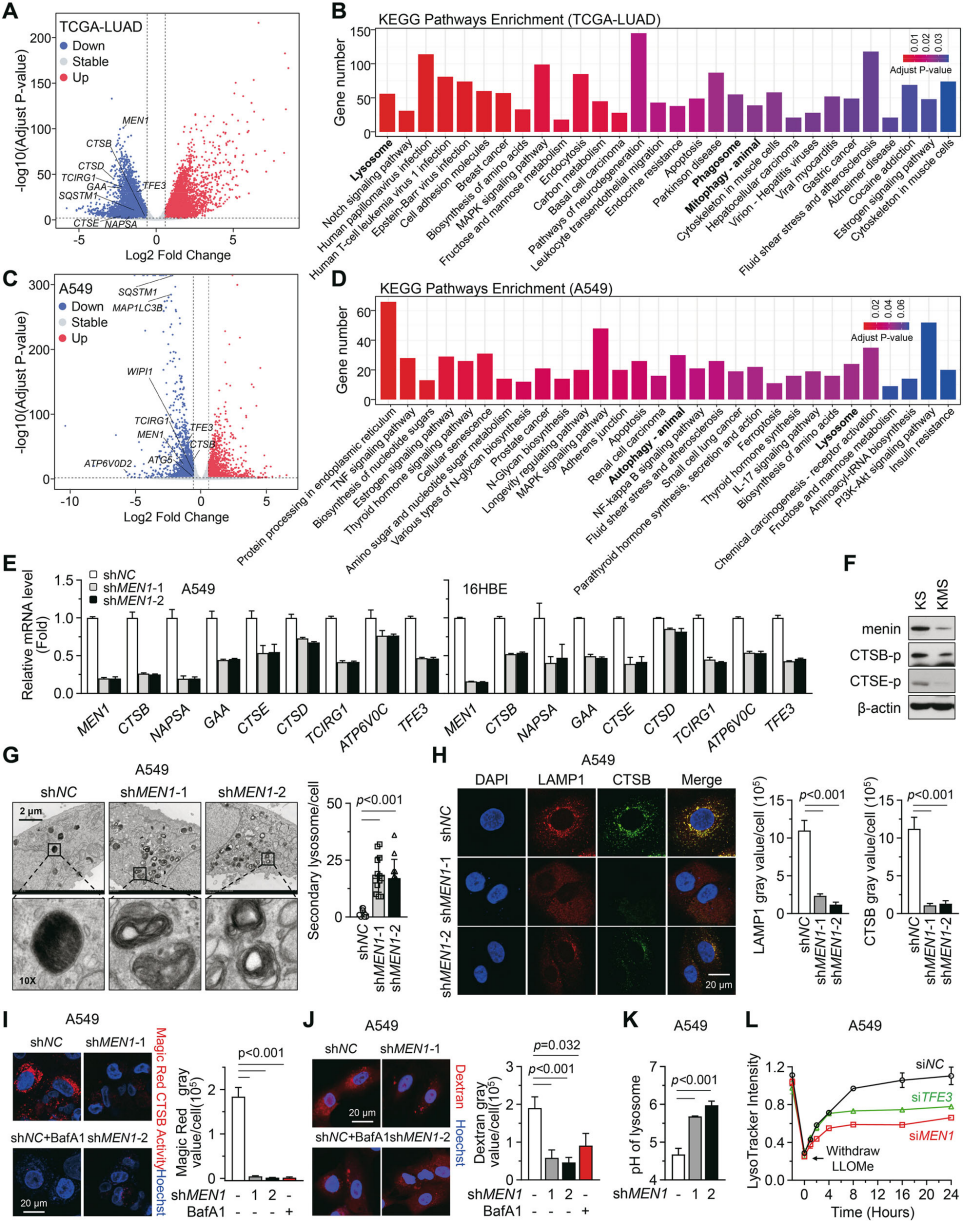
Menin maintains lysosomal enzyme expression and function. **A** Volcano plot showing 4685 downregulated and 4689 upregulated genes in the *MEN1*-Low group compared to the *MEN1*-High group. Some relevant genes were labeled in the plot. The selection criteria were an adjusted *P*-value < 0.01 and a fold-change <2/3 or >3/2. **B** Bar chart showing KEGG analysis of the downregulated genes in (**A**). **C** Volcano plot showing 1961 downregulated and 1842 upregulated genes in the *MEN1*-KD A549 cells compared to the control cells. Some relevant genes were labeled in the plot. The selection criteria were an adjusted *P*-value < 0.01 and a fold-change <2/3 or >3/2. **D** Bar chart showing KEGG analysis of the downregulated genes in (**C**). **E** RT-qPCR was performed to analyze the mRNA levels of indicated genes in A549 and 16HBE cells. All RT-qPCR results were normalized to *ACTB* mRNA levels. Fold-change values were calculated relative to the first column. The bar graph data represent the mean ± SD of three independent experiments. **F** Western blotting analysis of protein levels of menin, CTSB-p and CTSE-p in lung cancer tissues isolated from KS and KMS mice. **G** Transmission electron microscopy (TEM) was used to visualize the lysosomes in A549 cells, and their quantity was plotted as mean ± SD. The scale bar represents 2 μm, and a local area was magnified by 10 times. **H** Immunofluorescence (IF) staining of the LAMP1 (Red) and CTSB (Green) in A549 cells. DAPI visualizing nuclei. Scale bar, 20 μm. All the fluorescence intensity was quantified using ImageJ software. The bar graph represents the mean ± SEM. **I** CTSB enzymatic activity was determined by Magic Red staining (Red) in A549 cells. Hoechst visualizing nuclei. Scale bar, 20 μm. BafA1 (20 nM, 12 h) treatment serves as positive control. **J** Cellular endocytic activity was detected by Texas Red Dextran staining in A549 cells. Hoechst visualizing nuclei. Scale bar, 20 μm. BafA1 (20 nM, 12 h) treatment serves as positive control. **K** The pH of lysosomes was determined by calculating the fluorescence ratio of LysoSensor staining in A549 cells, and the results were plotted as the mean ± SD. **L** LysoTracker fluorescence intensity was used to detect intact lysosomes at different time points after withdrawing LLOMe. A549 cells were transfected with siRNA 3 days prior and then treated with 1 mM LLOMe for 2 h to disrupt lysosomal membranes.

We analyzed transcriptomic data from 537 lung adenocarcinoma specimens (TCGA-LUAD) in The Cancer Genome Atlas (TCGA). The samples were divided into two groups based on *MEN1* expression levels (*MEN1*-Low and *MEN1*-High), and samples with intermediate expression levels (Gray) were excluded from the analysis (Supplementary Fig. S1A). We found that some lysosomal genes were significantly lower in the *MEN1*-low group compared with those in the *MEN1*-High group (Fig. 1A; Supplementary Table S1). Kyoto Encyclopedia of Genes and Genomes (KEGG) analysis revealed significant correlations between *MEN1* expression and lysosome pathways (Fig. 1B). To assess whether menin regulates the transcription of lysosomal genes, we performed an RNA-Seq assay in A549 cells. The results indicated that *MEN1*-KD obviously downregulated certain lysosomal genes, including *ATP6V0D2* and *CTSB* (Fig. 1C; Supplementary Table S2). KEGG analysis also revealed a correlation between the lysosome pathway and *MEN1*, although this pathway did not rank among the top (Fig. 1D). Additionally, transcriptomic analysis of both TCGA-LUAD and A549 cells revealed significant correlations between *MEN1* and cell adhesion molecules (Adherens junctions in A549 cells), as well as with the mitogen-activated protein kinase (MAPK) signaling pathway (Fig. 1B, D). These findings are consistent with our previous reports that menin tightly regulates lung cancer cell migration and proliferation [18, 19], indicating the validity of our transcriptomic analysis. Western blotting analysis showed that *MEN1* shRNA-knockdown (KD) noticeably inhibited the expression of both the precursor (p) and mature (m) forms of CTSB and CTSE in the lung cancer cell lines (A549 and NCI-H157) and the immortalized bronchial epithelial cell line (16-HBE) (Supplementary Fig. S1B). The downregulation of lysosomal genes (including *CTSB*, *CTSE*, and alpha glucosidase (*GAA*)) by *MEN1*-KD was confirmed by RT-qPCR (Fig. 1E; Supplementary Fig. S1C, *p* < 0.05).

To further ascertain the role of menin in regulating the transcription of lysosomal genes in lung cancer, we established a genetically engineered mouse model (GEMM) with genotypes including wild-type (WT), alveolar type II cell (ATII)-specific *Men1* knockout (KO) (*Men1*^*f/f*^; *Sftpc-Cre* (MS)), ATII-specific *Kras*^*G12D*^ mutation (*LSL-Kras*^*G12D/+*^; *Sftpc-Cre* (KS)), and ATII-specific *Kras*^*G12D*^ mutation combined with *Men1* KO (*LSL-Kras*^*G12D/+*^; *Men1*^*f/f*^; *Sftpc-Cre* (KMS)) [9]. The protein expression of CTSB and CTSE in lung cancer tissues from the KMS group was found to be lower than that in the KS group, as evidenced by western blotting and immunohistochemistry (IHC) analyses (Fig. 1F; Supplementary Fig. S1D). Meanwhile, in the lung cancer tissues of the KMS group, the mRNA levels of lysosomal genes like *Ctsb*, *Ctse*, and *Gaa* were also lower in comparison to those in the KS group (Supplementary Fig. S1E, *p* < 0.05). These results indicate that the transcription of specific lysosomal genes was regulated by menin in KRAS^G12D^-driven lung cancer. Notably, between MS and WT, there was no significant difference in the expression of CTSB and CTSE in lung tissues (Supplementary Fig. S1D). To test whether menin regulates lysosomal genes only in the presence of KRAS-activating mutations, we isolated primary Mouse Embryonic Fibroblasts (MEFs) with the genotypes of WT, *Men1*^*∆/∆*^, *Kras*^*G12D/+*^, and *Kras*^*G12D/+*^; *Men1*^*∆/∆*^. RT-qPCR results showed that *Men1*^*∆/∆*^ did not inhibit the transcription of *Ctsb*, *Gaa*, and *Tfe3*, compared to WT mice (Supplementary Fig. S1F, p < 0.05). However, *Kras*^*G12D/+*^; *Men1*^*∆/∆*^ obviously counteracted the upregulation of *Ctsb*, *Ctse*, *Gaa*, and *Tfeb3* induced by *Kras*^*G12D/+*^ (Supplementary Fig. S1F). Moreover, in reisolated primary MEFs and ATII cells without *Kras* mutant, *Men1*-KO did not reduce the mRNA levels of many lysosomal genes (Supplementary Fig. S1G, *p* < 0.05). These results suggest that menin is a novel regulator of lysosomal gene expression, especially in cell with KRAS mutant.

We further investigated whether menin maintains the lysosomal function. The morphology of lysosomes often reflects their function. Transmission electron microscopy (TEM) revealed that the lysosomes with irregular shapes and containing incompletely degraded substrates, defined as secondary lysosomes, were significantly accumulated in *MEN1*-KD cells (Fig. 1G). Immunofluorescence (IF) staining showed that *MEN1*-KD reduced the puncta of LAMP1 and CTSB (Fig. 1H; Supplementary Fig. S1H, I). These results indicate that the loss of menin leads to a reduction in healthy lysosomes. Additionally, the enzymatic activity of CTSB was assessed using the Magic Red CTSB activity assay. An inhibitor, Bafilomycin A1 (BafA1), was used as a positive control, which specifically inhibits lysosomal acidification. The result showed that the enzymatic activity of CTSB was significantly inhibited by menin downregulation (Fig. 1I). Moreover, the Dextran staining indicated that the lysosome-associated endocytosis was impaired by *MEN1*-KD (Fig. 1J). The LysoSensor probe labelling revealed that the lysosomal pH was also elevated by *MEN1*-KD (Fig. 1K).

To investigate whether the loss of menin leads to disruptions in lysosomal repair or biogenesis, we designed a set of experiments using the LysoTracker probe and L-leucyl-L-leucine methyl ester hydrobromide (LLOMe) [20]. The LysoTracker probe labels intact lysosomes, and the probe signal significantly decreases when the lysosomal membrane is disrupted by LLOMe [20]. After treating cells with LLOMe for 2 hours, the removal of LLOMe led to a rapid recovery of the LysoTracker signal in the control cells (Fig. 1L; Supplementary Fig. S1J). However, *MEN1*-KD hindered the signal recovery to a greater extent than *TFE3*-KD (Fig. 1L; Supplementary Fig. S1J). Fluorescence staining also revealed that the LysoTracker signal was attenuated by *MEN1*-KD (Supplementary Fig. S1K). Altogether, our findings indicate that menin is a crucial regulatory factor in maintaining lysosomal gene expression, and loss of *MEN1* leads to severe lysosomal dysfunction.

### *MEN1* deletion inhibits autophagic flux

**Fig. 2:**
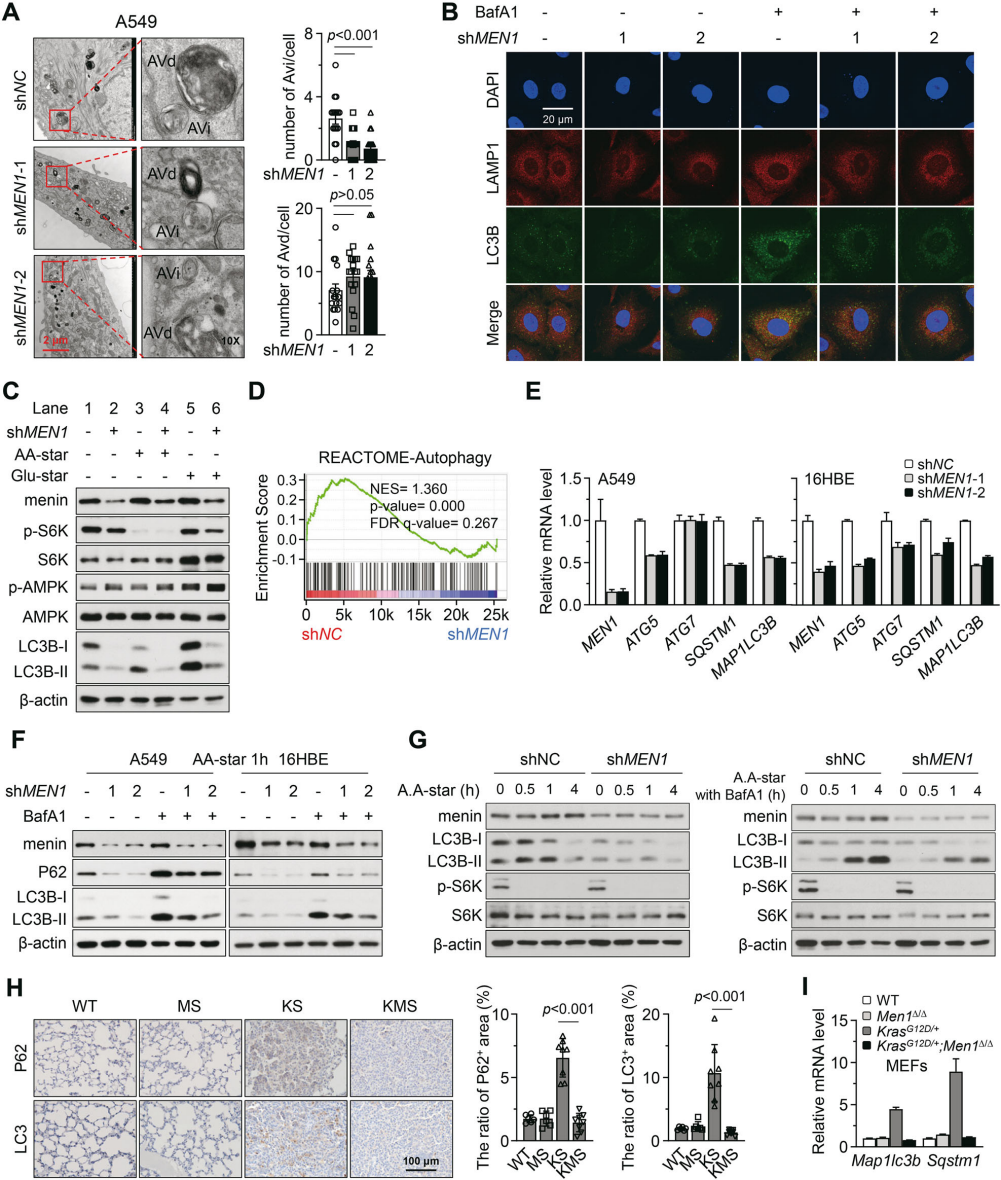
*MEN1* deletion inhibits autophagic flux. **A** TEM was used to visualize the Avi and Avd in A549 cells. The quantity was plotted as mean ± SEM. The scale bar represents 2 μm, and a local area was magnified by 10 times. **B** The A549 cells were treated with or without BafA1 (50 nM for 4 h), and IF staining were used to detect the expression of LAMP1 and LC3B. DAPI visualizing nuclei. Scale bar, 20 μm. **C** Western blotting was performed to assess the expression of indicated proteins under amino acid starvation (AA-star, 2 h) or glucose starvation (glu-star, 4 h) in A549 cells. **D** GSEA showing correlation between *MEN1* expression and the autophagic gene signature in the transcriptomic data of A549 cells. **E** RT-qPCR was performed to analyze the mRNA levels of indicated genes in A549 and 16HBE cells with *MEN1*-KD. **F** Western blotting was performed to assess the impact of *MEN1*-KD on the expression of indicated proteins in A549 and 16HBE cells. **G** Western blotting was performed to dynamically assess the impact of *MEN1*-KD on the expression of indicated proteins in A549 cells after AA-star and BafA1 treatment. **H** IHC staining was performed for the indicated proteins in the mouse lung tissues from WT (*n* = 6), MS (*n* = 6), KS (*n* = 8), and KMS (*n* = 8). The scale bar represents 100 μm. The positive area was quantified using ImageJ software and plotted as mean ± SD. **I** RT-qPCR was performed to analyze the mRNA levels of indicated genes in MEFs isolated from WT, *Men1*^*∆/∆*^, *Kras*^*G12D/+*^ and *Kras*^*G12D/+*^; *Men1*^*∆/∆*^ mice.

The completion of autophagic flux relies on intact lysosomal function [11]. Autophagosome (initial autophagic vacuole, Avi) is a type of vesicular structure with a double-layered or multilayered membrane that encloses cytoplasmic components during the early stages of autophagic flux [11]. The autophagosome fuses with the lysosome to form an autolysosome (degradative autophagic vacuoles, Avd), a single-layered membrane vesicular structure containing degraded cytoplasmic components, to complete the autophagic flux [11]. Therefore, impairment of lysosomal function leads to the accumulation of autophagosomes and autolysosomes, resulting in a blockage of autophagic flux. To assess whether the loss of menin leads to autophagic flux blockade, we examined the quantity of autophagosomes and autolysosomes in cells under amino acid starvation (AA-star) using TEM. The results revealed that *MEN1*-KD did not significantly promote the accumulation of autolysosomes; interestingly, it inhibited the quantity of autophagosomes (Fig. 2A). To further confirm the inhibition of autophagosome formation by *MEN1*-KD, we detected LC3B expression. During the process of autophagosome formation, the free form LC3B-I aggregates on the autophagosome membrane and transforms into LC3B-II, which appears as puncta in IF [11]. IF analysis showed that the LC3B puncta were reduced by *MEN1*-KD, and this reduction was further amplified by the lysosomal inhibitor BafA1 (Fig. 2B; Supplementary Fig. S2A, *p* < 0.05). The formation of autophagosome is promoted by AMPK pathway activation or mTORC1 pathway suppression [11]. We further assessed whether menin regulates autophagosome through these pathways. Western blotting analysis showed that AA-star inhibited the phosphorylation of ribosomal S6 kinase (p-S6K) at Thr389, a substrate of mTORC1, and increased LC3B-II level (Fig. 2C, lane 3). The glucose starvation (glu-star) activated the phosphorylation of AMPKα1/2 (p-AMPK) at Thr183/Thr172 and LC3B-II conversion (Fig. 2C, lane 5). However, *MEN1*-KD did not promote p-S6K or inhibit p-AMPK (Fig. 2C, lane 2), despite the downregulation of LC3B-II (Fig. 2C, lanes 4 and 6). These results indicate that menin regulates autophagosomes in a manner independent of mTORC1 and AMPK.

Through the transcriptome analysis of TCGA-LUAD and A549 cells, we found that *MEN1* is significantly correlated with the transcription levels of certain autophagy genes (Figs. 1A–D, 2D; Supplementary Table S3). RT-qPCR confirmed that shRNA-mediated *MEN1*-KD suppressed the expression of important autophagic genes, such as *ATG5*, *SQSTM1*, and *MAP1LC3B*, in A549 and 16-HBE cells (Fig. 2E, *p* < 0.05). Neither AA-star nor glu-star reversed the downregulation of autophagic genes caused by *MEN1*-KD (Supplementary Fig. S2B, *p* < 0.05). Autophagic genes such as *SQSTM1* and *MAP1LC3B* are crucial for maintaining the autophagic flux [11]. Western blotting showed that KD of *MEN1* markedly reduced the basal levels of LC3B-II and the accumulations of LC3B-II induced by BafA1 (Fig. 2F). Due to the significant inhibition of *SQSTM1* transcription by *MEN1*-KD, the protein levels of P62 were downregulated, both with and without BafA1 treatment (Fig. 2F). *MEN1*-KD also inhibited the mRNA levels of *ATG5*, *SQSTM1*, and *MAP1LC3B* and decreased the protein levels of P62 and LC3B-II in another lung cancer cells (Supplementary Fig. S2C, *p* < 0.05). Additionally, *MEN1*-KD inhibited the conversion of LC3B-II at multiple time points during AA-star, with or without BafA1 (Fig. 2G). Transient KD of *MEN1* via siRNA also inhibited the accumulation of P62 and LC3B-II proteins induced by BafA1 (Supplementary Fig. S2D). These results indicate that menin regulates autophagic flux by modulating the expression of key autophagy effector molecules. In vivo, IHC staining showed that *Men1*-KO inhibited the expression of P62 and LC3 in KRAS^G12D^-induced lung cancer (Fig. 2H). Moreover, *Men1*-KO markedly suppressed the transcription of *Map1lc3b* and *Sqstm1* in primary MEFs with *KRAS*^*G12D*^ mutant (Fig. 2I, *p* < 0.05).

It has been reported that the PI3K-AKT and MAPK-ERK pathways regulate AMPK-mTORC1 signaling during oncogene- or cytokine-induced autophagy [21]. We thus investigated whether menin-regulated autophagic flux is associated with these classical pathways. Consistent with our previous finding [19], *MEN1-*KD activated the p-AKT and p-ERK pathways (Supplementary Fig. S2E). However, *MEN1*-KD activated p-AMPK, which is a signal promoting autophagy, and did not consistently regulate p-S6K, an autophagy-inhibiting signal (Supplementary Fig. S2E). We further investigated whether menin relies on the PI3K-AKT or MAPK-ERK pathways to regulate autophagic molecules. U0126 (MAPK inhibitor) and LY294002 (PI3K-AKT inhibitor) failed to rescue the reduced LC3B-II caused by the *MEN1*-KD (Supplementary Fig. S2F). Furthermore, the expression of menin was not affected by AA-star, glu-star, or treatment with rapamycin (mTORC1 inhibitor), despite significant changes in CTSB (Supplementary Fig. S2G).

### Menin maintains lysosomal gene expression through H3K4me3

**Fig. 3:**
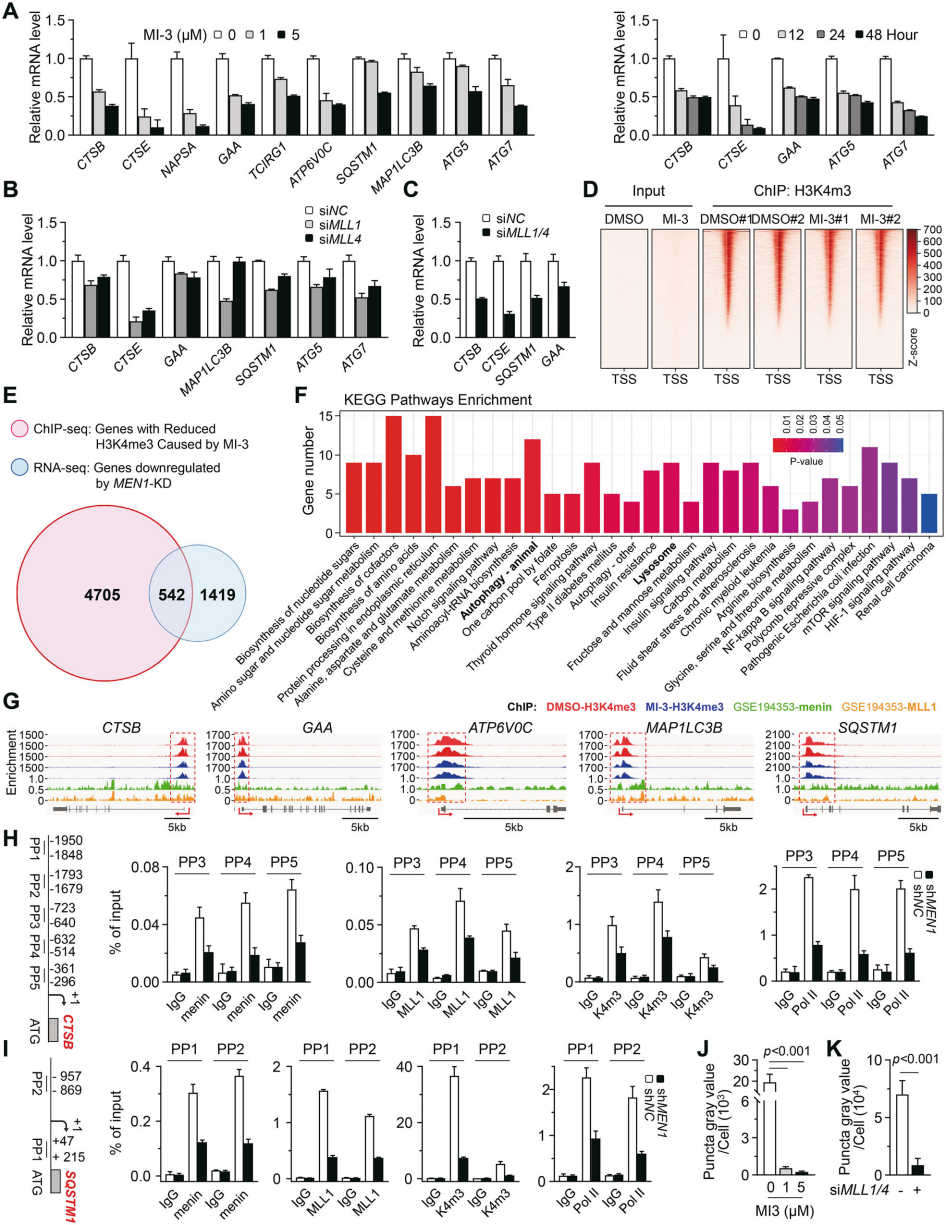
Menin maintains lysosomal gene expression through H3K4me3. **A** RT-qPCR was performed to analyze the mRNA levels of the indicated genes in A549 cells. The cells treated with different doses of MI-3 for 24 h or treated with 1 μM MI-3 for 12-48 h. **B** RT-qPCR was performed to analyze the mRNA levels of indicated genes in A549 cells with *MLL1*-KD or *MLL4*-KD. **C** RT-qPCR was performed to analyze the mRNA levels of indicated genes in A549 cells with simultaneous KD of *MLL1* and *MLL4* by siRNA. **D** Heatmap showing the coverage profiles for H3K4me3 within a 5 kb range of the transcriptional start site (TSS). **E** Venn diagram showing the overlap between genes with reduced H3K4me3 at TSSs upon MI-3 exposure and genes downregulated by *MEN1*-KD. **F** Bar chart showing KEGG analysis of the overlapping genes in (**E**). **G** Diagrams presenting the genome browser view of normalized ChIP-seq signals of H3K4me3, menin, and MLL1 for target genes. **H**, **I** Diagrams showing the primer pairs (PPs) designed for ChIP targeting the promoter regions of *CTSB* and *SQSTM1*. ChIP-qPCR was performed with antibodies of anti-menin, anti-H3K4me3, anti-MLL1 and anti-RNA polymerase II in A549 cells. IgG served as the negative control. **J** The bar graph representing the fluorescence quantification of Magic Red staining for CTSB enzymatic activity. The A549 cells were treated with indicated doses of MI-3 for 48 h. **K** The bar graph representing the fluorescence quantification of LysoTracker staining. The A549 cells were treated with siRNAs simultaneously targeting *MLL1* and *MLL4* for 3 days.

MLL-mediated H3K4me3 remodeling is an important epigenetic mechanism for menin-mediated regulation of gene transcription [6]. We asked whether this mechanism is essential for menin to regulate the lysosomal and autophagic genes. MI-3, an inhibitor that specifically disrupts the interaction between menin and MLL, inhibits chromatin H3K4me3 modification and gene transcription [22]. Here, we found that exposure to MI-3 dose-dependently decreased the H3K4me3 levels and the protein expression of CTSB and P62 (Supplementary Fig. S3A). RT-qPCR further revealed that MI-3 inhibited the expression of lysosomal and autophagic genes in a dose- and time-dependent manner (Fig. 3A, *p* < 0.05). MI-3 also inhibited autophagic flux demonstrated by the downregulation of LC3B-II (Supplementary Fig. S3B). Furthermore, siRNA-mediated KD of *MLL1* or *MLL4* inhibited lysosomal and autophagic gene expression (Fig. 3B; Supplementary Fig. S3C, *p* < 0.05). Simultaneous KD of *MLL1* and *MLL4* strongly inhibited the transcription of lysosomal genes (Fig. 3C, *p* < 0.05) and BafA1-induced accumulation of LC3B-II (Supplementary Fig. S3D). These results indicate that menin relies on MLL to regulate the transcription of certain lysosomal and autophagic genes.

To demonstrate the regulation of menin-mediated H3K4me3 on the transcription of specific lysosomal and autophagic genes, we performed chromatin immunoprecipitation sequencing (ChIP-seq) for H3K4me3 in A549 cells. We observed clear peaks of H3K4me3 around the transcription start sites (TSSs) (Fig. 3D), indicating that H3K4me3 modifications are present in a wide range of promoters. The H3K4me3 levels at the TSSs of 5,247 genes were reduced by exposure to MI-3 (Fig. 3E). Among them, a total of 542 genes were downregulated by *MEN1*-KD (Fig. 3E). The KEGG analysis of these overlapping genes showed that the lysosome and autophagy pathways were enriched (Fig. 3F). Moreover, exposure to MI-3 reduced H3K4me3 at the TSSs of certain lysosomal and autophagic genes, including *CTSB*, *GAA*, *SQSTM1*, and *MAP1LC3B* (Fig. 3G). In addition, analysis of a set of recent ChIP-seq data (GSE194353) in A549 cells from the Gene Expression Omnibus (GEO) also revealed that menin and MLL1 are co-enriched at the TSSs of these genes (Fig. 3G). To confirm these results, we conducted a series of ChIP experiments. The ChIP-qPCR primers targeting the promoters of *CTSB*, *CTSE*, *MAP1LC3B* and *SQSTM1* were designed (Fig. 3H, I; Supplementary Fig. S3E, G). The results revealed that menin clearly bound to these promoters, accompanied by the binding of MLL1 and enrichment of H3K4me3 (Fig. 3H, I; Supplementary Fig. S3E–H). Furthermore, KD of *MEN1* reduced the binding of MLL1 and RNA polymerase II and the enrichment of H3K4me3 at these promoter sites (Fig. 3H, I; Supplementary Fig. S3E–H). These results suggest that menin-mediated H3K4me3 is an epigenetic mechanism regulating the transcription of certain lysosomal and autophagic genes.

The histone modification H3K36me3, which also promotes gene transcription [23], was used as a negative control in our experiment. The H3K36me3 inhibitor sinefungin (SNF) clearly decreased the overall levels of H3K36me3 but did not affect the expression of *CTSB* and *CTSE* (Supplementary Fig. S3I, J). Although the overall H3K36me3 was significantly activated by the agonist JIB-04, it had no effect on *CTSB* and *CTSE* expression (Supplementary Fig. S3K, L). Another specific H3K36me3 inhibitor EZM0414 also did not affect the transcription of the lysosomal and autophagic genes (Supplementary Fig. S3M, N). ChIP assays revealed a weak enrichment of H3K36me3 at the promoter region of *CTSB*, and it was not significantly affected by *MEN1*-KD (Supplementary Fig. S3O).

In primary ATII cells isolated from GEMMs, *Mll1*-KO inhibited the expression of *Ctsb*, *Ctse*, *Sqstm1* and *Map1lc3b* compared to that in WT mice, and KO of either *Mll1* or *Men1* even more dramatically inhibited the expression of these genes in cells with *Kras*^*G12D*^ mutation (Supplementary Fig. S3P). In lysosomal function assessment, we observed that exposure to MI-3 clearly inhibited lysosomal CTSB activity in A549 cells (Fig. 3J; Supplementary Fig. S3Q). LysoTracker staining was also reduced by *Mll1-KO* in MEFs or *MLL1*/*MLL4*-KD in A549 cells (Fig. 3K; Supplementary Fig. S3R). These results further confirmed that menin regulates the transcription of certain lysosomal and autophagic genes through a mechanism of MLL-mediated H3K4me3. The transcriptions of lysosomal and autophagic genes are activated in response to nutrient deprivation [10]. However, AA-star did not enhance the binding of menin or the enrichment of H3K4me3 at the *CTSB* promoter (Supplementary Fig. S3S), suggesting that the regulation of lysosomal genes by menin is independent of the classical mTORC1 pathway. In summary, these findings demonstrate that menin-MLL mediated H3K4me3 remodeling is an important epigenetic mechanism in maintaining the expression of certain genes related to lysosome and autophagy.

### Menin epigenetically regulates TFE3

**Fig. 4:**
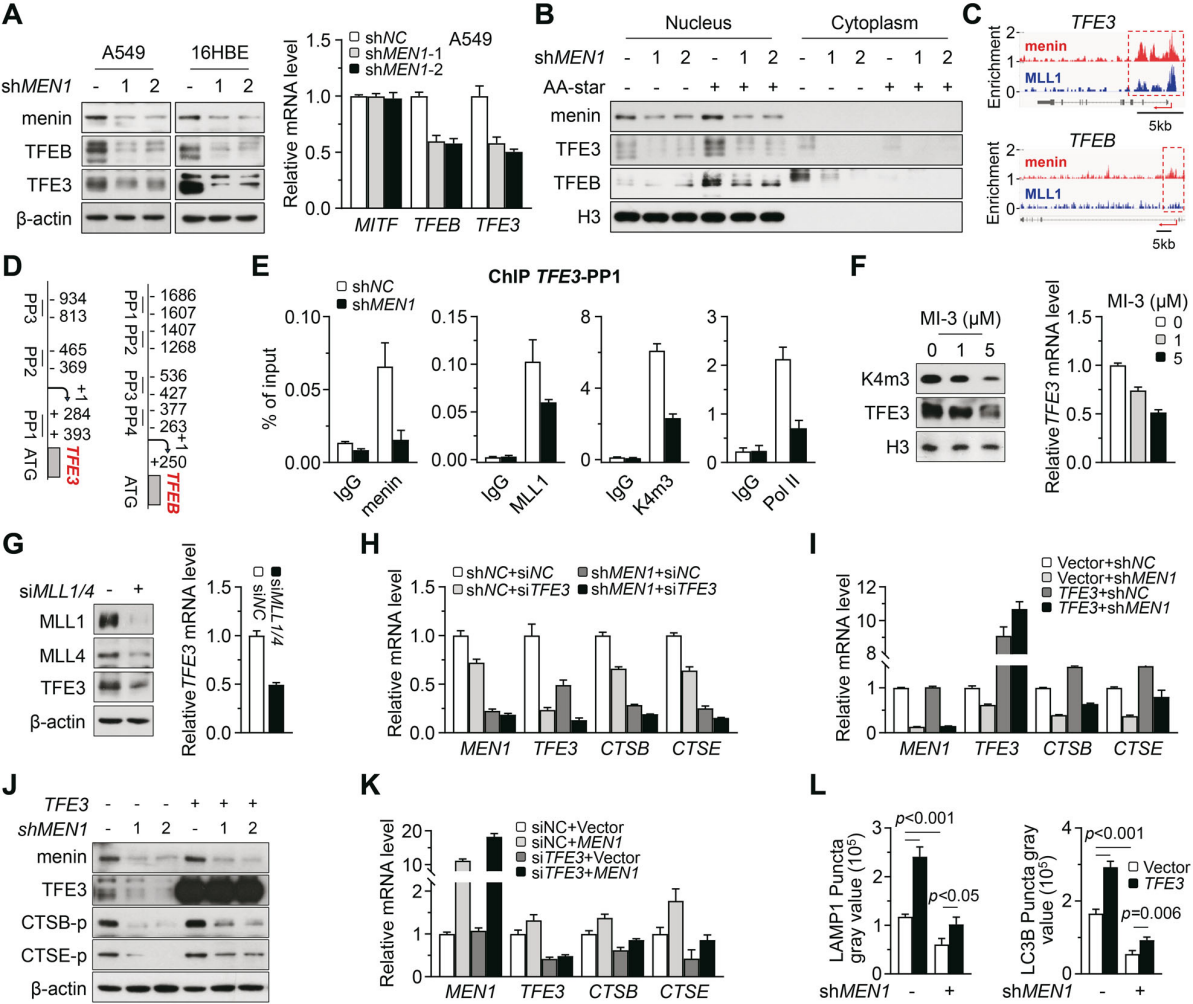
Menin epigenetically regulates TFE3. **A** Western blotting was used to assess the expression of indicated proteins in A549 and 16HBE cells (left). RT-qPCR was employed to measure mRNA levels of the corresponding genes in *MEN1*-KD A549 cells (right). **B** The A549 cells were exposed to AA-star for 2 h, and the nuclear and cytoplasmic proteins were extracted. Western blotting was used to examine the localization of TFE3 and TFEB in the nucleus and cytoplasm. H3 was used as the loading control. **C** Diagrams showing the genome browser view of normalized menin and MLL1 ChIP-seq signals for target genes. **D** Diagrams showing the PPs designed for ChIP targeting the promoter regions of *TFEB* and *TFE3* genes. **E** The ChIP-qPCR for *TFE3* was performed with antibodies of anti-menin, anti-H3K4me3, anti-MLL1 and anti-RNA polymerase II in control and *MEN1*-KD A549 cells. IgG served as the negative control. **F** Western blotting and RT-qPCR were used to examine the TFE3 expression in A549 cells exposure to MI-3 for 3 days. **G** The A549 cells were transfected with siRNAs simultaneously targeting *MLL1* and *MLL4*. After 3 days, western blotting and RT-qPCR were used to examine the expression of *TFE3*. **H**, **I** The *MEN1*-KD A549 cells were transfected with either siRNA-*TFE3* or full-length *TFE3*. RT-qPCR was used to measure the mRNA levels of the indicated genes. **J** The *MEN1*-KD A549 cells were transfected with full-length *TFE3*. Western blotting was used to examine the expression of indicated proteins. **K** The stable *MEN1*-overexpression A549 cells were transfected with siRNA-*TFE3*. RT-qPCR was used to measure the mRNA levels of the corresponding genes. **L** The *MEN1*-KD A549 cells were transfected with full-length *TFE3*. Bar graph was generated to quantitatively analyze the fluorescence intensity of LAMP1 and LC3B in IF experiments. Values were presented as mean ± SEM.

TFE3 and TFEB are two important transcription factors for directly controlling the expression of lysosomal and autophagic genes [10]. We further investigated whether TFE3 and TFEB patriciate in menin-mediated transcription of lysosomal genes. Co-immunoprecipitation (Co-IP) experiments revealed that menin did not interact with TFE3 and TFEB under physiological conditions or under AA-star stress (Supplementary Fig. S4A). However, the mRNA and protein levels of TFE3 and TFEB were clearly reduced by menin deficiency (Figs. 1E, 4A; Supplementary Fig. S1E, F, *p* < 0.05). The protein levels of TFE3 and TFEB in the nucleus were also decreased by *MEN1*-KD, especially under AA-star stress (Fig. 4B).

The analysis results of the ChIP-seq data (GSE194353) showed that menin and MLL were clearly co-enriched at the TSSs of *TFE3*, while the enrichments at the TSSs of *TFEB* were very weak (Fig. 4C). Then, we designed ChIP-qPCR primers targeting the promoter regions of *TFE3* and *TFEB* (Fig. 4D) and performed the ChIP-assay. The results also revealed that menin strongly bound to the promoter region of *TFE3* but not to that of *TFEB* (Supplementary Fig. S4B). Furthermore, *MEN1*-KD clearly reduced the binding of MLL1 and RNA polymerase II at the *TFE3* promoter, as well as the enrichment of H3K4me3 (Fig. 4E). Simultaneous KD of *MLL1* and *MLL4* also markedly inhibited the enrichment of H3K4me3 at the *TFE3* promoter region (Supplementary Fig. S4C). Disruption of the menin-MLL interaction by exposure to MI-3 led to a downregulation of TFE3 expression, dose-dependently (Fig. 4F, *p* < 0.05). KD of *MLL1* and *MLL4*, either individually or simultaneously, also effectively suppressed TFE3 expression (Fig. 4G; Supplementary Fig. S4D, *p* < 0.05). These findings indicate that menin regulates the transcription of *TFE3* through MLL-mediated H3K4me3.

Due to the fact that TFE3 serves as a critical transcription factor for lysosomal and autophagic genes [10], menin may indirectly regulate the transcription of these genes by modulating the expression of TFE3. Indeed, we observed that *TFE3*-KD exacerbated the reduction in the transcription of *CTSB* and *CTSE* caused by *MEN1*-KD (Fig. 4H). The overexpression of full-length *TFE3* partially mitigated this reduction (Fig. 4I, J). Furthermore, *TFE3*-KD inhibited the upregulation of *CTSB* and *CTSE* induced by menin-overexpression (Fig. 4K). This indirect regulatory mechanism is also reflected in LysoTracker staining, as the overexpression of *TFE3* partially restored the weak staining caused by *MEN1*-KD (Supplementary Fig. S4E, F). Similarly, the overexpression of *TFE3* partially restored the reduced puncta of LAMP1 and LC3B caused by *MEN1*-KD in A549 cells during AA-star (Fig. 4L; Supplementary Fig. S4G). These results suggest that menin’s transcriptional regulation of lysosomal genes partially depends on its transcriptional control of *TFE3*. AA-star stress did not promote the binding of menin or the enrichment of H3K4me3 at the *TFE3* promoter (Supplementary Fig. S4H), indicating that menin regulates *TFE3* in an mTORC1-independent manner. However, the regulatory mechanism of menin on *TFEB* is currently unclear. We observed that neither knockdown nor overexpression of *TFEB* affected the regulation of *CTSB* or *CTSE* by menin (Supplementary Fig. S4I–K).

### Loss of menin causes mitochondrial dysfunction

**Fig. 5:**
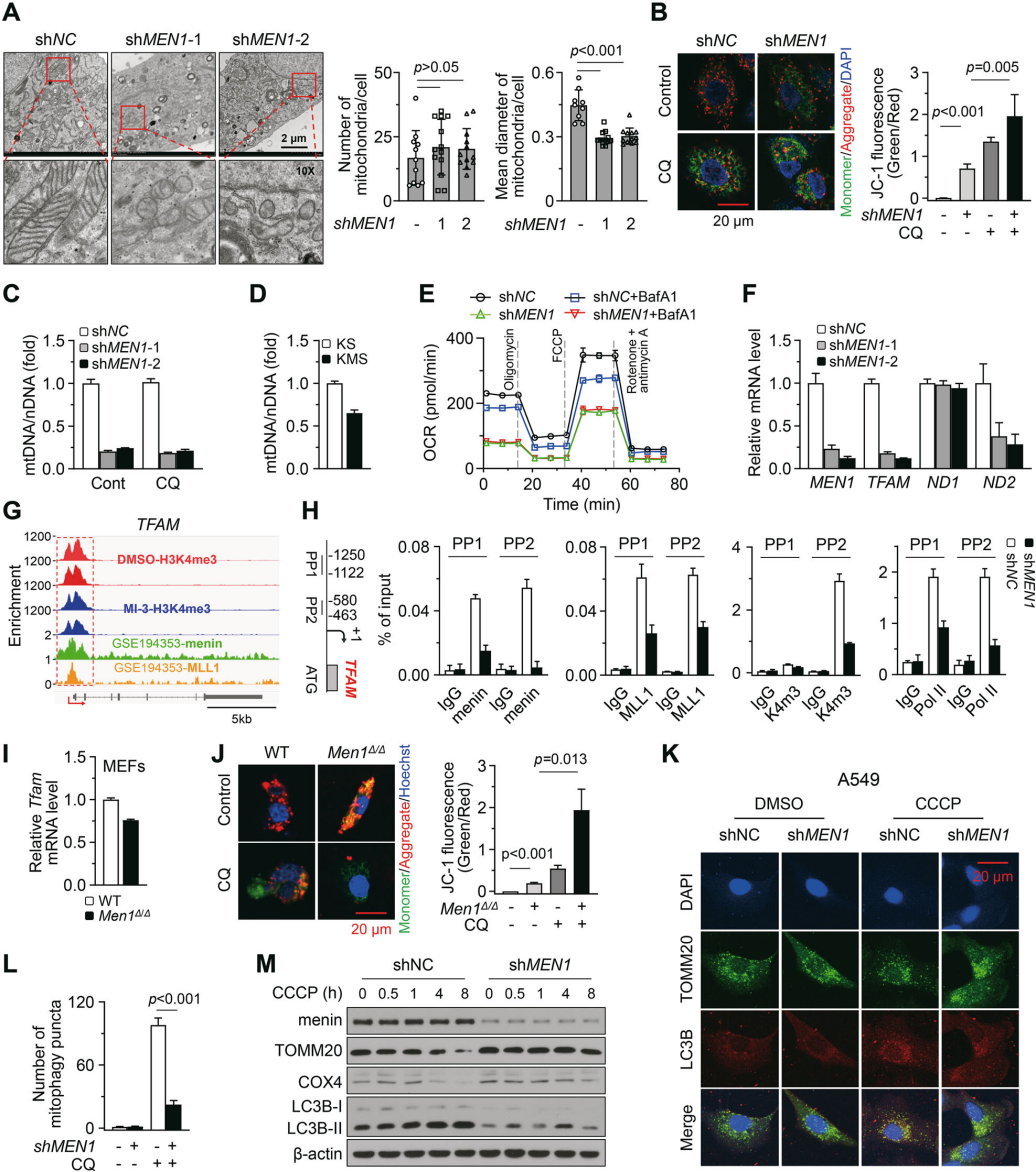
Loss of menin causes mitochondrial dysfunction. **A** TEM was employed to visualize the morphology of mitochondria in *MEN1*-KD A549 cells. The number and diameter of mitochondria were quantified and presented as mean ± SD. The scale bar represents 2 μm, and a specific region was magnified 10 times. **B** Control and *MEN1*-KD A549 cells were treated with or without CQ (10 μM, 12 h). JC-1 staining was used to detect membrane potential status of mitochondria. The fluorescence intensity was quantified, and the ratio of green to red was presented as the mean ± SEM. Hoechst visualizing nuclei. **C** Control and *MEN1*-KD A549 cells were treated with or without CQ (10 μM, 12 h). RT-qPCR was used to measure the mitochondrial mass by quantifying the expression of mtDNA (*ND1* gene) relative to nDNA (*HB2M* gene). The fold-change values were presented as mean ± SD. **D** Lung cancer tissues were isolated from KS and KMS mice. RT-qPCR was used to measure the mitochondrial mass by quantifying the expression of mtDNA (*Atp6* gene) relative to nDNA (*Tert* gene). **E** Control and *MEN1*-KD A549 cells were treated with or without BafA1 (20 nM, 12 h). The Seahorse Mito Stress Test was used to detect oxygen consumption rate in the indicated cells with the indicated inhibitors. **F** RT-qPCR was used to measure the mRNA levels of indicated genes in control and *MEN1*-KD A549 cells. **G** Diagrams showing the genome browser view of ChIP-seq signals of normalized H3K4me3, menin, and MLL1 for *TFAM*. **H** Diagram showing the PPs designed for ChIP targeting the promoter region of the *TFAM* gene. ChIP-qPCR was performed with antibodies of anti-menin, anti-H3K4me3, anti-MLL1 and anti-RNA polymerase II in control and *MEN1*-KD A549 cells. **I** RT-qPCR was used to measure the mRNA levels of *Tfam* in MEFs isolated from WT and *Men1*^*Δ/Δ*^ mice. **J** The indicated MEFs were treated with or without CQ (10 μM, 12 h). JC-1 staining was used to detect membrane potential status of mitochondria. **K**, **L** The *MEN1*-KD A549 cells were exposed to 20 μM CCCP (DMSO as control) for 4 h. IF staining of TOMM20 (Green) and LC3B (Red) in A549 cells. DAPI visualizing nuclei. Scale bar, 20 μm. All the co-localized fluorescence puncta of TOMM20 and LC3B were quantified using ImageJ software. The bar graph data represent the mean ± SEM. **M** Western blotting was performed to dynamically assess the impact of *MEN1*-KD on the expression of indicated proteins in A549 after CCCP (20 μM) exposure.

We are interested in investigating the biological effects of impaired lysosomal function and autophagy resulting from menin deficiency. Mitochondria, essential organelles in cells, have many crucial functions such as energy production and apoptosis regulation [12]. The fidelity of mitochondria relies heavily on the clearance mediated by autophagy and lysosomes, a process also known as mitophagy [11]. Despite not detecting mitochondria accumulation, we observed an obvious reduction in the cristae and volume of mitochondria in *MEN1*-KD A549 cells (Fig. 5A). Mitochondria with this morphology might have functional impairments. The oxidative phosphorylation function of mitochondria can be reflected by the mitochondrial membrane potential [12]. The JC-1 probe staining showed that *MEN1*-KD decreased the red fluorescence and increased the green fluorescence (Fig. 5B), indicating menin deficiency impaired oxidative phosphorylation function of mitochondria. Inhibiting of lysosomal function by Chloroquine (CQ) also significantly increased the green fluorescence intensity (Fig. 5B), indicating that the lysosomal dysfunction may further impair mitochondrial function. Mitochondrial DNA (mtDNA) integrity is fundamental for maintaining mitochondrial function, as it encodes the essential component of the oxidative phosphorylation [12]. We observed that the copies of mtDNA were greatly decreased by *MEN1*-KD (Fig. 5C, *p* < 0.05). Since the number of mitochondria was not influenced by *MEN1*-KD in the TEM images (Fig. 5A), we infer that the integrity of mtDNA is compromised due to menin deficiency. However, the copies of mtDNA remained unchanged following exposure to CQ (Fig. 5C), suggesting that menin maintains mtDNA integrity in a manner independent of lysosomal function. Additionally, mtDNA copies were also reduced in *Men1*-KO lung cancer tissues (Fig. 5D). The mitochondrial respiratory function can be reflected by the oxygen consumption rate (OCR) [13]. Seahorse mitochondrial stress assays showed that the OCR was inhibited by lysosome inhibitor BafA1 and further suppressed by *MEN1*-KD (Fig. 5E). These results suggest that menin also maintains mitochondrial function via mechanisms other than lysosomal function and autophagy.

In RNA-Seq analysis, we noted that *MEN1*-KD obviously suppressed the transcription of *TFAM* (Supplementary Fig. S5A; Supplementary Table S4), a gene essential for maintaining mtDNA integrity and mitochondrial function [24]. RT-qPCR confirmed that the expression of *TFAM* was significantly reduced by *MEN1*-KD (Fig. 5F; Supplementary Fig. S5B). In addition, TFAM expression was lower in tumor tissue from KMS mice compared to that from KS mice (Supplementary Fig. S5C). These results indicate that the transcription of *TFAM* is maintained by menin. Moreover, disruption of menin-MLL by MI-3 or *MLL1*/*MLL4*-KD inhibited TFAM expression in A549 cells (Supplementary Fig. S5D, E). Exposure to MI-3 also decreased the mtDNA copies dose-dependently (Supplementary Fig. S5F). Additionally, exposure to MI-3 or simultaneous KD of *MLL1* and *MLL4* reduced the OCR, while exposure to EZM0414 or *SETD2*-KD did not (Supplementary Fig. S5G). These results suggest that menin relies on MLL to maintain the transcription of *TFAM* and mitochondrial function. ChIP-seq analysis revealed that H3K4me3, menin, and MLL1 were co-enriched at the TSS of *TFAM*, and MI-3 decreased the H3K4me3 levels at this site (Fig. 5G). The ChIP-qPCR confirmed that menin bound to the *TFAM* promoter (Fig. 5H). Furthermore, *MEN1*-KD clearly inhibited the binding of MLL1 and RNA polymerase II, as well as the enrichment of H3K4me3 at these promoter sites (Fig. 5H). These results directly demonstrate that menin maintains the transcription of *TFAM* through MLL-mediated H3K4me3. In primary MEFs, menin also regulates the transcription of *Tfam* and mitochondrial function (Fig. 5I, J, *p* < 0.05). Altogether, our results suggest that menin regulates mitochondrial function through both a lysosome-dependent mechanism and direct regulation of *TFAM*.

Furthermore, the IF results showed that *MEN1*-KD led to a reduction in the puncta of the co-localization between LC3B and translocase of outer mitochondrial membrane 20 (TOMM20) in cells exposed to the mitophagy inducer CCCP (Fig. 5K, L). Western blotting revealed that *MEN1*-KD blocked the CCCP-induced degradation of TOMM20 and cytochrome c oxidase subunit 4 (COX4), and also restrained the elevation of LC3B-II (Fig. 5M). These results suggest that menin deficiency inhibits mitophagy. Recently, inhibitors disrupting the menin-MLL interaction have demonstrated promising therapeutic potential in preclinical studies and clinical trials for treating acute leukemia with MLL-AF fusions or NPM1 mutations [25]. Therefore, we tested whether this type of inhibitor leads to potential side effects related to lysosomal and autophagic dysfunction during treating leukemia. RT-qPCR revealed that the novel menin-MLL inhibitor Revumenib did not inhibit the transcription of those lysosomal and autophagic genes in the leukemia cell lines of MV4-11 and THP-1 (Supplementary Fig. S5H). This result prompts that menin regulates lysosomal and autophagic genes in a cell-context-dependent manner. Indeed, in the hepatocellular carcinoma cell line HepG2, *MEN1*-KD did not inhibit the transcription of lysosomal and autophagic genes such as *CTSB* and *SQSTM1* (Supplementary Fig. S5I). In contrast, in the cholangiocarcinoma cell line SK-Hep1 and the malignant melanoma cell line A375, *MEN1*-KD inhibited the transcription of these genes (Supplementary Fig. S5J, K). The ChIP results from A549 cells showed that MI-3 obviously inhibited the enrichment of H3K4me3 at the promoters of lysosomal and autophagic genes such as CTSB (Supplementary Fig. S5L). However, no such result was observed in MV4-11 cells (Supplementary Fig. S5M). These results indicate that in some cells, the menin-MLL-mediated H3K4me3 modification is not necessary for maintaining the expression of lysosomal and autophagic genes.

### Loss of menin causes metabolite accumulation in lung cancer

**Fig. 6:**
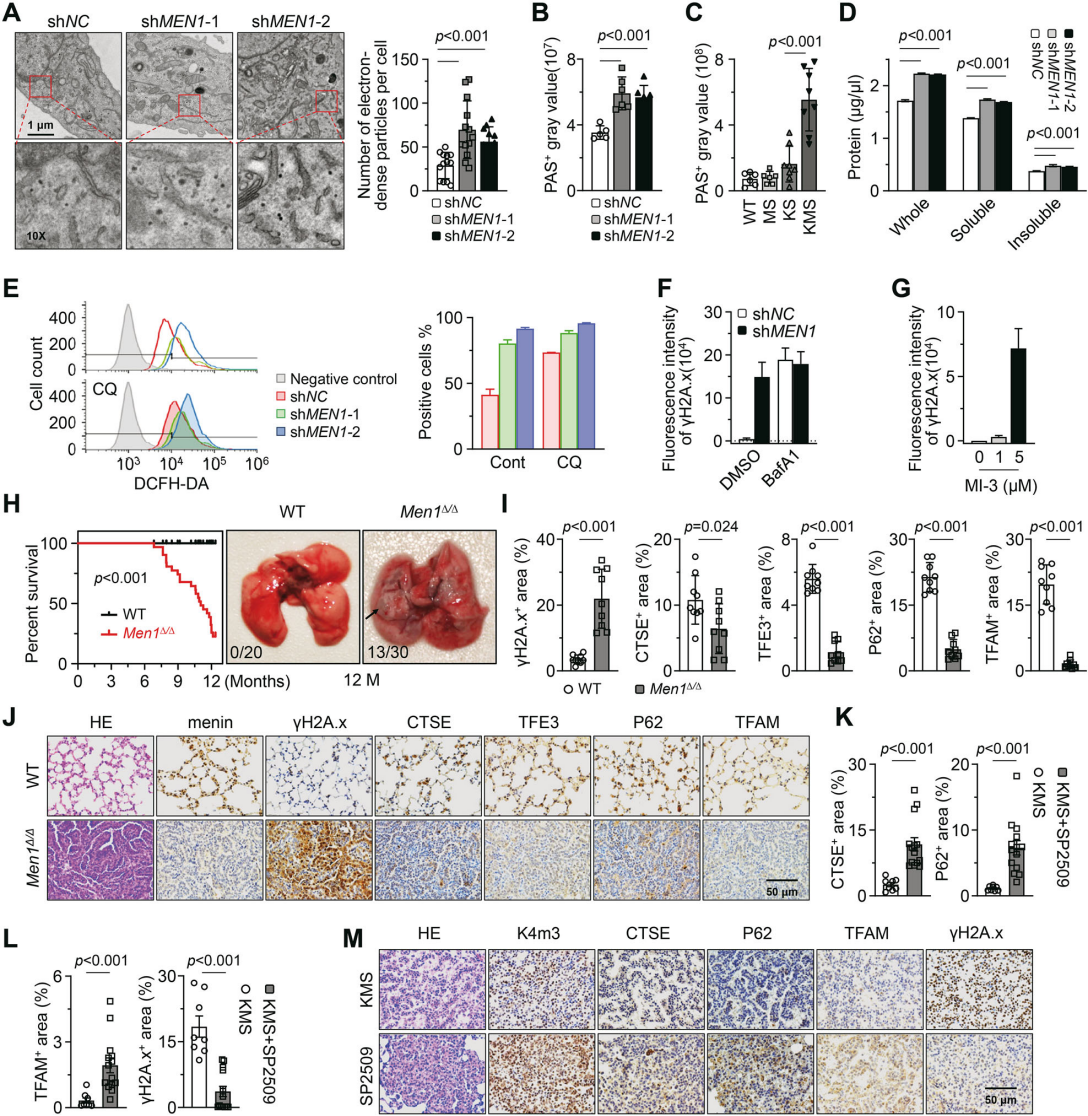
Loss of menin causes metabolite accumulation in lung cancer. **A** TEM was employed to visualize the electron-dense particles in control and *MEN1*-KD A549 cells. The number of the electron-dense particles was quantified and presented as mean ± SD. The scale bar represents 1 μm, and a specific region was magnified 10 times. **B** PAS staining was performed to detect glycogen in control and *MEN1*-KD A549 cells. The staining was quantified and presented as mean ± SD. **C** PAS staining was performed to detect glycogen in mouse lung tissues from WT (*n* = 6), MS (*n* = 6), KS (*n* = 8), and KMS (*n* = 8) at 2 months after TAM injection. The staining was quantified and presented as mean ± SD. **D** The whole, soluble, and insoluble proteins in A549 cells were determined by using the BCA kit. **E** Control and *MEN1*-KD A549 cells were treated with or without CQ (10 nM, 12 h). Flow cytometry was utilized to assess cellular ROS levels using the DCFH-DA probe (left panel). The cells without probe loading served as the negative control. The proportion of positive cells was presented as the mean ± SD (right panel). **F** Control and *MEN1*-KD A549 cells were treated with or without BafA1 (20 nM, 12 h). IF was performed to detect γH2A.x in indicated cells. The bar graph represents the mean ± SEM. **G** IF was performed to detect γH2A.x in A549 cells exposed to MI-3 (3 days). The bar graph represents the mean ± SEM. **H** The Kaplan-Meier survival curves, and representative dissection images of WT (*n* = 20) and *Men1*^*∆/∆*^ (*n* = 30) mice. **I** The quantification of IHC staining of indicated proteins in (**J**). The positive area was quantified and plotted as mean ± SD. **J** IHC staining was performed to detect the indicated proteins in the lung tissues from WT (*n* = 9) and *Men1*^*∆/∆*^ (*n* = 9) mice. Scale bars, 50 μm. **K**–**M** IHC staining was performed to detect the indicated proteins in the lung tissues from KMS mice with (*n* = 14) or without (*n* = 8) SP2509-treatment. The scale bar represents 50 μm. The positive area was quantified and plotted as mean ± SD.

Lysosomes are essential for the autophagic flux and are responsible for the degradation of a variety of biological macromolecules, including carbohydrates, proteins, and lipids [10]. TEM images showed that there was an obvious increase in electron-dense particles in *MEN1*-KD A549 cells (Fig. 6A). The previous RT-qPCR results showed that *MEN1*-KD inhibited the transcription of *GAA* (Fig. 1E), an important lysosomal enzyme responsible for breaking down glycogen [26]. Through Periodic acid-Schiff (PAS) staining, we confirmed that *MEN1*-KD led to glycogen deposition (Fig. 6B; Supplementary Fig. S6A). The glycogen in the lung cancer of KMS mice was also visibly higher than that in the mice of other genotypes (Fig. 6C; Supplementary Fig. S6B). Furthermore, there was a deposition of soluble and insoluble proteins in *MEN1*-KD A549 cells (Fig. 6D; Supplementary Fig. S6C). The Oil Red O staining showed that the lipid deposition was not induced by menin deficiency (Supplementary Fig. S6D, E). The reactive oxygen species (ROS) are primarily generated from mitochondria, and elevated ROS levels can lead to genomic instability, which is considered one of the important factors in tumorigenesis [12, 27]. Flow cytometry analysis showed that intracellular ROS levels were increased with either *MEN1*-KD or inhibition of lysosomal function (Fig. 6E). We then sought to determine whether DNA damage occurs in cells with menin deficiency. Consistently, obvious DNA damage was observed in A549 cells with *MEN1*-KD or after exposure to BafA1 (Fig. 6F; Supplementary Fig. S6F). Furthermore, disruption of the menin-MLL interaction by MI-3 also induced DNA damage (Fig. 6G; Supplementary Fig. S6G). These results suggest that menin deficiency further leads to the deposition of certain biological macromolecules in lung cancer.

We established the *Men1*^*f/f*^; *Ubc-Cre* (*Men1*^*Δ/Δ*^) GEMMs. After 7 months following tamoxifen injection, the *Men1*^*Δ/Δ*^ mice started to experience mortality, with a rate reaching 77.4% at 12 months (Fig. 6H). Moreover, the typical lung cancer morphology was observed in 13 out of the 30 *Men1*^*Δ/Δ*^ mice (Fig. 6H). Notably, in *Men1*-deletion induced tumors, lysosomal and mitochondrial proteins such as TFE3 and TFAM were downregulated, along with obvious DNA damage (Fig. 6I, J). We further aim to identify a drug to restore the expression of lysosomal, autophagic, and mitochondrial genes downregulated by menin deficiency. SP2509 is a specific antagonist of the histone demethylase LSD1 [16]. Previously, we found that SP2509 treatment reversed the downregulation of H3K4me3 caused by *Men1*-KO, thereby suppressing the development of KMS-driven lung cancer [9]. Here, SP2509 effectively restored the expression of genes such as *CTSE* and *TFAM* in the lung cancer of KMS mice, along with the restoration of H3K4me3 (Fig. 6K–M). Even the extent of DNA damage was alleviated by SP2509 (Fig. 6L, M). In lung cancer cell lines, we also observed that SP2509 restored the expression of CTSB, LC3B, and TFAM, which were suppressed by *MEN1*-KD (Supplementary Fig. S6H).

Finally, the P53-RB1 pathway, which has been reported to regulate lysosomal genes [28], is suppressed by menin deficiency [9]. We investigated whether menin’s regulation of lysosomal and mitochondrial genes is also dependent on this pathway. The results showed that *P53*-KD did not affect the regulatory effect of menin on the expression of CTSB and TFAM in A549 cells (Supplementary Fig. S6I). This suggests that menin regulates lysosomal and mitochondrial function in a manner independent of the P53-RB1 pathway.

## Discussion

**Fig. 7:**
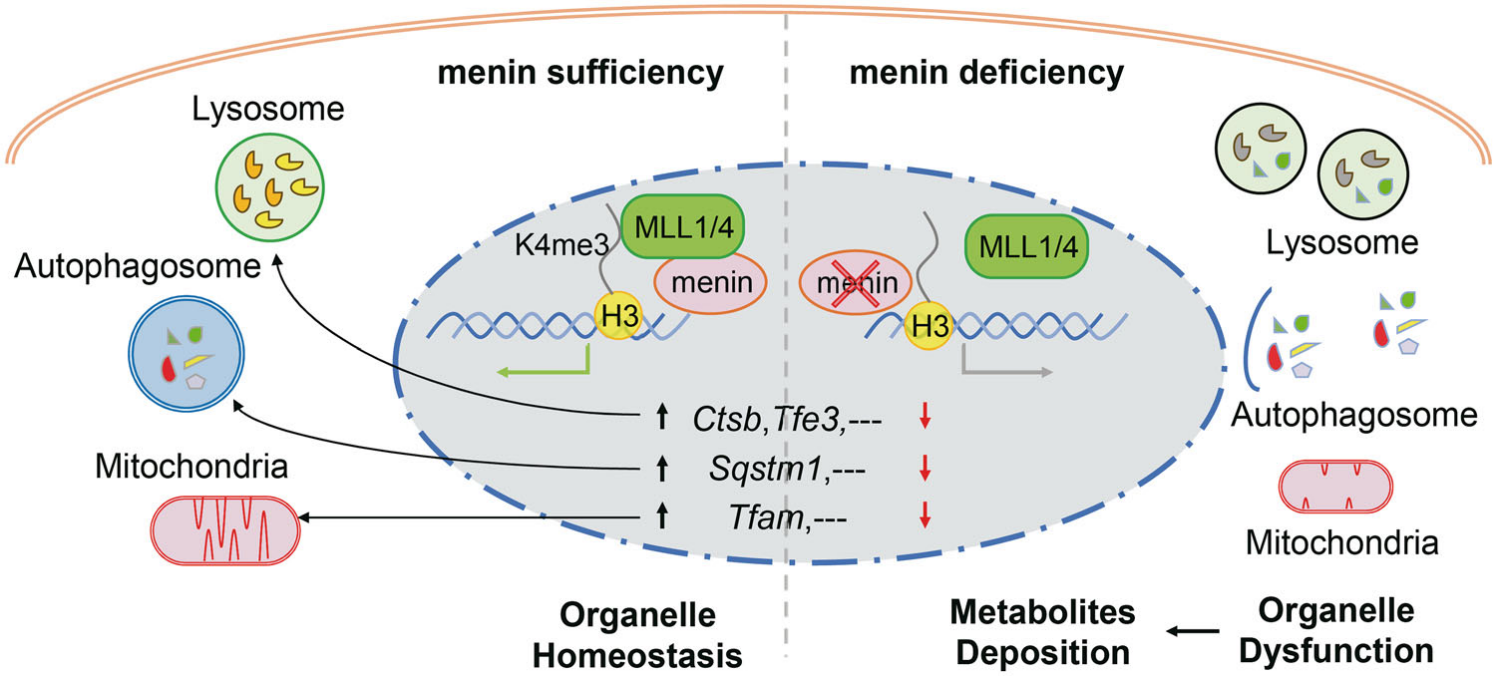
A model of the cellular mechanisms by which menin regulates organelle homeostasis.

To date, numerous studies have focused on the mechanism of lysosome and mitochondria activation under stress, but less attention has been given to the physiological regulatory mechanisms involved. Here, we demonstrate that menin is a novel transcriptional regulator that directly controls lysosomal and mitochondrial gene expression through an epigenetic mechanism. Although menin is widely involved in various histone modifications through its epigenetic role, the regulation of lysosomal and mitochondrial genes depends primarily on MLL-mediated H3K4me3 remodeling, which is necessary for lysosomal and mitochondrial biogenesis and homeostasis. Menin-MLL-regulated H3K4me3 remodeling is a conserved epigenetic mechanism that maintains organismal development and homeostasis, and its dysfunction can lead to various diseases [6]. For example, homozygous loss of *Men1* in mice results in embryonic lethality at embryonic day 11.5-13.5 and is associated with a variety of developmental defects [29]. Blocking menin/MLL expression or disrupting its interaction leads to the silencing of these lysosomal and mitochondrial genes, severely disrupting their homeostasis. However, this mechanism is not activated by stress conditions, such as AA-star, and does not rely on the classical mTORC1 and AMPK pathways. The exact experimental results indicate that another important positive histone modification, H3K36me3, does not participate in the transcriptional regulation of lysosomal and mitochondrial genes. These findings indicate that H3K4me3 remodeling could be one of the intrinsic cellular mechanisms through which menin-MLL maintains normal organismal development and homeostasis (Fig. 7A).

Here, menin was found to have a more pronounced regulatory effect on lysosomal and autophagic genes in cells with KRAS-activation. Abnormal activation of the RAS pathway reportedly has complex effects on the mTORC1 and AMPK pathways and ultimately activates the transcription of lysosomal and autophagic genes by promoting the nuclear location of TFEB and TFE3 [21]. It has been reported that KRAS-activation induces autophagic flux as a protective mechanism to adapt to the high levels of metabolic activity, thereby maintaining genomic stability by timely clearing metabolic byproducts [30]. Consequently, we postulate that the biological significance of KRAS-mediated activation of lysosomal function may be similar to that of cellular senescence, which serves as a mechanism suppressing tumor development [21, 31]. Our previous study has demonstrated that the expression of *MEN1* is inactivated in approximately 23-27% of lung cancer cases, and this inactivation is correlated with KRAS-mediated DNA methylation at the *MEN1* promoter region [9, 18]. Therefore, we propose that *MEN1* deficiency inhibits the lysosomal function upregulated by KRAS activation, leading to metabolite accumulation and genomic instability. This may also be one of the mechanisms by which *Men1* deficiency promotes KRAS-induced lung cancer [9]. Furthermore, we discovered that *MEN1* deficiency obstructed mitophagy. Compromised mitophagy fails to efficiently eliminate damaged mitochondria, thereby triggering oxidative stress and subsequently leading to genomic instability [12, 27]. These findings suggest that the normal functions of lysosomes and mitochondria, maintained by menin, are among the mechanisms of its tumor-suppressive activity (Fig. 7A).

In this study, we utilized multiple cell lines, including A549, NCI-H157, 16HBE, SK-Hep1, A375, Hep-G2, MV4-11, and THP-1, to analyze the regulation of lysosomal and mitochondrial genes by menin. These results suggest that menin regulates lysosomal and mitochondrial functions in a cell-specific manner. Why different cells have varying preferences for epigenetic mechanisms is an interesting question that needs further investigation. Additionally, we observed a significant correlation between menin and endoplasmic reticulum-related genes. The mechanisms by which menin regulates the endoplasmic reticulum and its biological significance also deserve in-depth study.

Overall, the present study revealed the previously unrecognized biological and mechanistic importance of menin-MLL-mediated H3K4me3 remodeling in maintaining organelle homeostasis.

## Materials and methods

### Mice

Animal welfare was ensured, and experimental procedures followed ethical regulations approved by the Institutional Animal Care Committee of Xiamen University. The mice were housed in a sterile room with controlled lighting, temperature, and humidity levels. We established the following genotypes of mice using the methods described previously [9, 32]: Wild-type (WT), *Men1*^*f/f*^; *Sftpc-Cre* (MS), *Mll1*^*f/f*^; *Sftpc-Cre* (MLS), *LSL-Kras*^*G12D/+*^; *Sftpc-Cre* (KS), *LSL-Kras*^*G12D/+*^; *Men1*^*f/f*^; *Sftpc-Cre* (KMS), and *LSL-Kras*^*G12D/+*^; *Mll1*^*f/f*^; *Sftpc-Cre* (KMLS); As well as *Men1*^*f/f*^; *Ubc-Cre* (*Men1*^*∆/∆*^), *LSL-Kras*^*G12D/+*^; *Ubc-Cre* (*Kras*^*G12D/+*^), *LSL-Kras*^*G12D/+*^; *Men1*^*f/f*^; *Ubc-Cre* (*Kras*^*G12D/+*^; *Men1*^*∆/∆*^), *Mll1*^*f/f*^; *Ubc-Cre* (*Mll1*^*∆/∆*^). The animal sources have been described in our previous studies [9, 32]. The sample size for animal experiments was determined by power analysis and adjusted based on pilot data and prior studies. Subsequently, animals were randomized into different groups. Upon reaching the age of 6-8 weeks, the mice with *Sftpc-Cre* or *Ubc-Cre* were administered intraperitoneal injections of tamoxifen (TAM) (Sigma-Aldrich, T5648) at a dosage of 100 mg/kg once daily for five consecutive days, followed by additional treatments. The TAM was dissolved in corn oil containing 10% ethanol. One week later, PCR analysis was conducted to verify the genotype using genotyping primer sequences synthesized by Sangon Biotech (Listed in Supplementary Table S5). In survival studies, the endpoints encompassed various indicators such as labored and rapid breathing, decreased food intake or mobility, loss of energy, or weight loss exceeding 20% of the initial body weight [9]. A pre-established criterion is that non-disease deaths of animals due to improper factors such as infighting among animals are excluded from experimental analysis. There is no blinding of researchers or participants.

### Chromatin immunoprecipitation (ChIP) assays

ChIP assays were performed following the protocol described in our previous report [33], with minor modifications. Approximately 2 million cells were used for ChIP assays. The cells were cross-linked with 1% formaldehyde at room temperature. For histone ChIP, the cross-linking time was 10 min, followed by sonication at 20% power for 2 min using a sonicator (SONICS, VCX150) with the samples immersed in an ice-water bath. For other protein ChIP, the cross-linking time was 15 min, followed by sonication at 30% power for 2 min. The corresponding antibodies (Listed in Supplementary Table S6; 1:300) were incubated with the samples overnight at 4 °C. The remaining steps were carried out according to the protocol provided by the ChIP Assay kit (Millipore, 17-295). The DNA was purified using phenol-chloroform extraction and quantified by RT-qPCR. The primer sequences used for the ChIP assays are listed in Supplementary Table S5.

### ChIP-seq analysis

The preparation of samples for ChIP-seq is similar to that of ChIP assays. Specifically, the sonication conditions were adjusted to 20% power for 3 min, which facilitates obtaining DNA fragments within the range of 100 bp to 500 bp. The purified DNA samples were then sent to Azenta Life Sciences (Suzhou, China) for subsequent procedures. Two biological replicates were conducted for ChIP-seq. Next generation sequencing library preparations were constructed following the manufacturer’s protocol. Subsequently, these libraries were sequenced using an Illumina NovaSeq 6000 instrument according to manufacturer’s instructions (Illumina, San Diego, CA, USA). MACS (2.1.1) software was employed with the mapped reads to identify the statistically significant ChIP-enriched peaks in comparison to the corresponding input group. Differentially enriched peaks were identified based on a fold-change > 2 and *P*-value < 0.001. All regions were annotated with respect to the gene whose transcriptional start sites (TSS) were closest to the center of the peak region. Gene visualization was performed using the Integrative Genomics Viewer (2.19.1).

### Seahorse Mito Stress Test

The Seahorse XF Cell Mito Stress Test Kit (Agilent, 103015-100) was utilized for conducting the mitochondrial stress test. Measurements were performed using the XFe/XF96 Analyzer (Agilent, 102416-100) to determine the oxygen consumption rate (OCR). The cells were seeded at a density of 10,000/well in a 96-well Seahorse plate. Three biological replicates were carried out for each experimental group. All procedures were performed according to the manufacturer’s protocol. The final concentrations of reagents were as follows: 1 μM of oligomycin (Agilent, 103591-100), 2 μM of FCCP (Selleck, S8276), and 0.5 μM of antimycin A/rotenone (Agilent, 103591-100) in each experimental group.

### Transmission electron microscope (TEM) experiment

TEM assays were performed following the protocol described in a previous report [34]. Briefly, the cells were collected and fixed overnight in 2.5% glutaraldehyde solution. Subsequent procedures were carried out by the Electron Microscopy Facility at Xiamen University, using an electron microscope (ThermoFisher, Helios 5 UC) to capture images.

### LysoTracker staining

To assess the lysosomal repair capacity, the experiments were performed according to the report with minor modifications [20]. Corresponding cells were seeded in light-protected 96-well plates (Beyotime, FCP965). A concentration of 1 mM LLOMe was used to disrupt the lysosomal membrane. LysoTracker (Beyotime, C1046) was used at a concentration of 50 nM for labelling of lysosomes. The fluorescence values were collected using a fluorescence plate reader. For direct observation of lysosomes, corresponding cells were seeded in glass-bottom culture dishes (Beyotime, FCFC020). The LysoTracker staining was performed as described above, and the cells were simultaneously incubated with 5 μM Hoechst dye (Abcam, ab228551). The images were acquired using an inverted fluorescence microscope. Magic Red CTSB activity (Abcam, ab270772) and Texas Red dextran (Invitrogen, D1830) staining resembled those of LysoTracker.

### LysoSensor

To measure the lysosomal pH, the experiments were conducted based on a previous report with minor modifications [35]. Corresponding cells were seeded in light-protected 96-well plates and treated with 50 μg/mL of the LysoSensor probe (Invitrogen, L22460). The fluorescence microplate reader was set to an excitation wavelength of 360 nm, and emission signals were recorded at wavelengths of 460 nm and 540 nm. The 460/540 fluorescence emission ratios were used to calculate lysosomal pH according to a calibration curve. Calibration buffers of known pH were used to prepare a calibration curve with cells treated with the LysoSensor probe.

### JC-1 staining

Corresponding cells were seeded in glass-bottom culture dishes. JC-1 (Beyotime, C2003S) working solution was prepared according to the manufacturer’s instructions. Images were acquired using an inverted fluorescence microscope. The software ImageJ was used to quantify the red and green fluorescence intensities, and the ratio of green to red fluorescence was calculated to assess the extent of mitochondrial membrane potential depolarization.

### ROS Flow cytometry analysis

The cells were collected and washed with PBS (Servicebio, G4202) prior to DCFH-DA (Beyotime, S0033S) incubation. DCFH-DA was diluted in serum-free culture medium to achieve a final concentration of 10 μM. After collecting the cells, they were suspended in the diluted DCFH-DA solution at a density of one million cells per milliliter and incubated at 37 °C for 20 min. The cells were then washed three times with serum-free cell culture medium. The proportion of positive cells was detected using a flow cytometer (Beckman, CytoFlex S).

### Statistics and reproducibility

Sample size was calculated using power analysis and then adjusted based on pilot data and prior studies. All statistical analyses were conducted using GraphPad Prism (9.0.0) software. Student’s *t*-test (two-tailed) was used to compare statistical significance between two groups. Kaplan–Meier method was used to establish overall survival curves, and the log-rank test was used to compare significant differences. The variation within each group of data was estimated. The variances between groups were found to be similar and conformed to the corresponding statistical tests. *P*-values < 0.05 were considered significant. Each experiment was performed in triplicate or more. The meanings of all center values and error bars are presented in each figure legend.

## Supplementary information

Supplementary Figures

Supplementary Tables

Supplementary Materials and Methods

Supplemental Material-Original western blotting bands

Reproducibility checklist

## Data Availability

The sequencing data generated in this study have been deposited in the Gene Expression Omnibus database under accession code GSE272371 and GSE285593. The original western blotting bands are included in *Supplemental Material-Original western blotting bands*. All other original data that support the findings of this study are available from the corresponding author upon reasonable request.

## Code availability

The data analysis code used in this paper is available at the following repository: [https://github.com/junboy123/MEN1].
