# Supplementary material for: Menin maintains lysosomal and mitochondrial homeostasis through epigenetic mechanisms in lung cancer
Source: Cell Death Dis. 2025 Mar 8;16(1):163. doi: 10.1038/s41419-025-07489-0 (PMC11890858; doi:10.1038/s41419-025-07489-0)
Supplement: Supplementary file 1 — Supplementary Figures [file 41419_2025_7489_MOESM1_ESM.docx]

**Supplementary Figure S1-S6**





**Supplementary Figure S1.** Menin maintains lysosomal enzyme expression and function. (**A**) Histogram showing the distribution of *MEN1* expression levels in the transcriptomic data of 537 cases from TCGA-LUAD. Based on this, *MEN1*-Low (n = 19) and *MEN1*-High (n = 413) transcriptomic data were selected for further analysis. (**B**) Western blotting analysis was used to detect the expression of the precursor (p) and mature (m) forms of CTSB and CTSE in cells with *MEN1*-knockdown (KD) mediated by shRNA. β-actin served as the loading control. (**C**) RT-qPCR was performed to analyze the mRNA levels of indicated genes in NCI-H157 cells with shRNA-mediated *MEN1*-KD. The shRNA of sh*MEN1*-1 was used in this experiment. (**D**) The images showing the staining of HE and IHC of the lung tissues from Wild type (WT, n = 6), *Men1^f/f^*; *Sftpc-Cre* (MS, n = 6), *LSL-Kras^G12D/+^*; *Sftpc-Cre* (KS, n = 8), and *LSL-Kras^G12D/+^*; *Men1^f/f^*; *Sftpc-Cre* (KMS, n = 8). IHC staining was performed for the indicated proteins. The scale bar represents 100 μm. The positive area was quantified and plotted as mean ± SD. (**E**) RT-qPCR was performed to analyze the mRNA levels of indicated genes in lung cancer tissues from KS and KMS. (**F**) RT-qPCR was performed to analyze the mRNA levels of indicated genes in MEFs isolated from GEMMs with the following genotypes: WT, *Men1^∆/∆^*, *Kras^G12D/+^* and *Kras^G12D/+^*; *Men1^∆/∆^*. (**G**) RT-qPCR was performed to analyze the mRNA levels of indicated genes in MEFs isolated from GEMMs with genotype WT or *Men1^∆/∆^*, and ATII cells isolated from WT or MS. (**H**, **I**) IF showing the expression and co-localization of LAMP1 and CTSB in NCI-H157 cells. The fluorescence intensity was quantified and plotted as mean ± SEM. DAPI visualizing nuclei. Scale bar, 20 μm. (**J**) Western blotting was used to detect the expression of menin and TFE3 in A549 cells with siRNA-mediated *MEN1*-KD or *TFE3*-KD. (**K**) LysoTracker staining in A549 cells. Hoechst visualizing nuclei. Scale bar, 20 μm.





**Supplementary Figure S2.** *MEN1* deletion inhibits autophagic flux. (**A**) The A549 cells were treated with or without BafA1 (50 nM for 4 h), and IF staining was used to detect the expression and co-localization of LAMP1 and LC3B. The fluorescence intensity was quantified and plotted as mean ± SD. (**B**) RT-qPCR was performed to analyze the mRNA levels of indicated genes in A549 cells under AA-star or glu-star. The shRNA of sh*MEN1*-1 was used in this experiment. (**C**) RT-qPCR and western blotting were performed to analyze mRNA and protein expression of indicated genes in *MEN1*-KD NCI-H157 cells. For the western blotting, the cells were treated with or without BafA1 (50 nM, 4 h). The shRNA of sh*MEN1*-1 was used in these experiments. (**D**) Western blotting was performed to assess the expression of LC3B and P62 proteins in siRNA-mediated *MEN1*-KD A549 cells. (**E**) Western blotting was used to assess the expression of indicated proteins in *MEN1*-KD cell lines. (**F**) The control and *MEN1*-KD A549 cells were treated with MEK inhibitor (U0126) or PI3K inhibitor (LY294002) at 5 μM for 48 h, and western blotting was used to assess the expression of indicated proteins. All groups received a treatment of BafA1 (50 nM) for 4 h. GAPDH served as the loading control. (**G**) Western blotting was performed to detect the expression of indicated proteins. The A549 cells were treated with AA-star or glu-star for different times, as well as indicated concentrations of Rapamycin for 36 h.





**Supplementary Figure S3.** Menin maintains lysosomal gene expression through H3K4me3. (**A**) Western blotting was used to examine the expression of indicated proteins in A549 cells exposed to indicated doses of MI-3 (3 days). H3 served as the loading control. (**B**) The A549 cells were treated with MI-3 and/or BafA1, and western blotting was used to examine the expression of indicated proteins. (**C**) Western blotting was used to examine the KD effects of siRNAs targeting *MLL1* or *MLL4* in A549 cells. (**D**) Western blotting was performed to assess the impact of simultaneous KD of *MLL1/4* on the expression of indicated proteins. (**E**, **F**) Diagrams showing the primer pairs (PPs) designed for ChIP targeting the promoter regions of *CTSE*. ChIP-qPCR was performed using antibodies of anti-menin, anti-H3K4me3, anti-MLL1 and anti-RNA polymerase II in A549 cells. IgG served as the negative control. (**G**, **H**) Diagrams showing the primer pairs (PPs) designed for ChIP targeting the promoter regions of *MAP1LC3B*. ChIP-qPCR was performed using antibodies of anti-menin, anti-H3K4me3, anti-MLL1 and anti-RNA polymerase II in A549 cells. IgG served as the negative control. (**I**) Western blotting was used to examine the expression of indicated proteins in A549 cells exposed to indicated doses of SNF for 3 days. (**J**) RT-qPCR was utilized to examine the mRNA expression of *CTSB* and *CTSE* in A549 cells exposed to indicated doses of SNF for 3 days. (**K**, **L**) Western blotting and RT-qPCR were employed to investigate the impact of JIB-04 on the protein and mRNA expression of the indicated genes in A549 cells. (**M**, **N**) Western blotting and RT-qPCR were utilized to examine the expression of indicated genes in A549 cells exposed to indicated doses of EZM0414 for 3 days. (**O**) ChIP-qPCR was performed with anti-H3K36me3 antibody in A549 cells. IgG served as the negative control. The shRNA of sh*MEN1*-1 was used in these experiments. (**P**) RT-qPCR was employed to assess the mRNA levels of the indicated genes in ATII cells isolated from mice with the following genotypes: WT, *Mll1^f/f^*; *Sftpc-Cre* (MLS), MS, KS, *LSL-Kras^G12D/+^*; *Mll1^f/f^*; *Sftpc-Cre* (KMLS), and KMS. (**Q**) Magic Red staining for CTSB enzymatic activity was performed on A549 cells treated with indicated doses of MI-3 for 48 h. Hoechst visualizing nuclei. Scale bar, 20 μm. (**R**) The LysoTracker staining was performed on A549 cells treated with siRNAs targeting *MLL1*/*4* for 3 days and on MEFs with *Mll1*-KO. The bar graph representing the fluorescence quantification of MEFs. (**S**) ChIP-qPCR for *CTSB* was performed with anti-menin and anti-H3K4me3 antibodies in A549 cells with or without AA-star. IgG served as the negative control.





**Supplementary Figure S4.** Menin epigenetically regulates TFE3. (**A**) The 293T cells were transfected with Flag-*MEN1*, HA-*TFE3*, and either His-*TFEB* or HA-*TFEB*. Co-IP was conducted to analyze the potential interaction between menin and TFE3 or between menin and TFEB. All overexpressed plasmids contained the full-length coding sequences of the respective genes and were tagged accordingly. (**B**) ChIP-qPCR was performed with anti-menin antibody. IgG served as the negative control. ChIP-PPs of *TFE3* or *TFEB* were used. (**C**) ChIP-qPCR was performed with anti-H3K4me3 antibody in *MLL1/4*-KD A549 cells. (**D**) RT-qPCR was used to measure the mRNA levels of *TFE3* in A549 cells with siRNA-mediated *MLL1*-KD or *MLL4*-KD. (**E**, **F**) LysoTracker staining was performed on A549 cells with *MEN1*-KD and full-length *TFE3* overexpression. Hoechst visualizing nuclei. Fluorescence intensity was quantified and presented as mean ± SEM. The shRNA of sh*MEN1*-1 was used in these experiments. (**G**) IF experiment was performed on A549 cells with *MEN1*-KD and full-length *TFE3* overexpression. These cells underwent amino acid starvation (containing 50 nM BafA1) for 4 h prior to the IF experiment. Anti-LAMP1 and anti-LC3B antibodies were used in this experiment, and DAPI was used to visualize nuclei. (**H**) ChIP-qPCR was performed with anti-menin and anti-H3K4me3 antibodies in A549 cells with or without AA-star. (**I**, **J**) The *MEN1*-KD A549 cells were transfected with either siRNA-*TFEB* or full-length *TFEB*. RT-qPCR was used to measure the mRNA levels of indicated genes. (**K**) The A549 cells with stable *MEN1* overexpression were transfected with siRNA-*TFEB*. RT-qPCR was used to measure the mRNA levels of indicated genes.





**Supplementary Figure S5.** Loss of menin causes mitochondrial dysfunction. (**A**) GSEA analysis showing correlation between *MEN1* expression and the mitochondrial gene signature in RNA-Seq data of A549 cells. The shRNA of sh*MEN1*-1 was used in this experiment. (**B**) Western blotting was used to examine the expression of TFAM in control and *MEN1*-KD A549 cells. β-actin served as the loading control. (**C**) Western blotting and RT-qPCR were used to examine the expression of indicated genes in lung cancer tissues isolated from KS and KMS mice. (**D**) Western blotting and RT-qPCR were used to examine the expression of TFAM in A549 cells exposed to indicated doses of MI-3 for 3 days. (**E**) RT-qPCR was used to measure the mRNA levels of *TFAM* in *MLL1*-KD or *MLL4*-KD A549 cells. (**F**) A549 cells were treated with MI-3 for 3 days. RT-qPCR was used to measure the mitochondrial mass by quantifying the expression of mtDNA (*ND1* gene) relative to nDNA (*HB2M* gene). The fold-change values were presented as mean ± SD. (**G**) A549 cells were treated with DMSO, MI-3 or EZM0414 for 3 days (left panel). A549 cells were transfected with siRNAs targeting *MLL1*/*4* or *SETD2* for 3 days (right panel). The Seahorse Mito Stress Test was used to detect oxygen consumption rate in the indicated cells. The bars represent mean ± SD. (**H**) RT-qPCR was used to measure the mRNA levels of indicated genes in MV4-11 and THP-1 cells exposed to Revumenib (3 days). (**I-K**) RT-qPCR was used to measure the mRNA levels of indicated genes in HepG2, SK-Hep1 and A375 cells. (**L**, **M**) ChIP-qPCR was conducted using an anti-H3K4me3 antibody in A549 and MV4-11 cells treated with MI-3 (5μM). ChIP-qPCR primers for the genes of *CTSB*, *TFE3*, *MAP1LC3B*, *SQSTM1*, and *TFAM* were used.





**Supplementary Figure S6.** Loss of menin causes metabolite accumulation in lung cancer. (**A**) PAS staining was performed to detect glycogen in control and *MEN1*-KD A549 cells. Scale bars, 50 μm. (**B**) PAS staining was performed to detect glycogen in lung tissues from WT (n = 6), MS (n = 6), KS (n = 8), and KMS (n = 8) mice, 2 months after TAM injection. Scale bars, 200 μm. (**C**) Coomassie Brilliant Blue staining was used to observe the concentrations of whole protein, soluble protein, and insoluble protein in control and *MEN1*-KD A549 cells. (**D**) Oil Red O staining was performed to detect lipid in lung tissues from WT (n = 6), MS (n = 6), KS (n = 8), and KMS (n = 8) mice, 2 months after TAM injection. Scale bars, 50 μm. The positive area was quantified and plotted as mean ± SD. (**E**) Oil red O staining was performed to detect lipoid in control and *MEN1*-KD A549 cells. The positive area was quantified and plotted as mean ± SD. (**F**) Control and *MEN1*-KD A549 cells were treated with or without BafA1 (20 nM, 12 h). IF was performed to detect γH2A.x in indicated cells. The shRNA of sh*MEN1*-1 was used in this experiment. (**G**) IF was performed to detect γH2A.x in A549 cells exposed to MI-3 (3 days). (**H**) The control and *MEN1*-KD A549 cells were treated with SP2509 (5 μΜ) for 48 h, and western blotting was used to assess the expression of indicated proteins. (**I**) P53 was knocked down by siRNA in A549 cells with *MEN1* overexpression or *MEN1*-KD. Western blotting was used to assess the expression of indicated proteins.
