# Supplementary material for: Menin maintains lysosomal and mitochondrial homeostasis through epigenetic mechanisms in lung cancer
Source: Cell Death Dis. 2025 Mar 8;16(1):163. doi: 10.1038/s41419-025-07489-0 (PMC11890858; doi:10.1038/s41419-025-07489-0)
Supplement: Supplementary file 2 — Supplementary Tables [file 41419_2025_7489_MOESM2_ESM.docx]

**Supplementary Table S1-S6**

**Supplementary Table S1**. The expression changes of lysosomal genes (KEGG-lysosome) in *Men1*-Low compared to *Men1*-High from TCGA-LUAD data.

| Gene | Fold Change | p-adj |
| --- | --- | --- |
| *CTSD* | 0.15 | 1.22E-21 |
| *ARSA* | 0.19 | 5.95E-04 |
| *CTSE* | 0.21 | 9.56E-36 |
| *GAA* | 0.21 | 8.88E-10 |
| *TCIRG1* | 0.22 | 1.59E-02 |
| *CLTB* | 0.24 | 6.88E-65 |
| *IDUA* | 0.25 | 3.32E-12 |
| *NAGLU* | 0.27 | 3.46E-47 |
| *AP3B2* | 0.28 | 8.28E-21 |
| *AP1B1* | 0.31 | 2.42E-03 |
| *NAPSA* | 0.31 | 1.07E-08 |
| *MCOLN1* | 0.32 | 3.20E-01 |
| *ABCA2* | 0.32 | 2.73E-06 |
| *SMPD1* | 0.32 | 8.29E-05 |
| *NAGPA* | 0.33 | 2.86E-28 |
| *CTSF* | 0.34 | 8.89E-02 |
| *GNPTG* | 0.35 | 4.90E-02 |
| *CTSA* | 0.35 | 3.98E-05 |
| *CTSH* | 0.35 | 3.55E-01 |
| *CTSZ* | 0.35 | 1.77E-01 |
| *AP1M2* | 0.36 | 3.25E-29 |
| *LAMP1* | 0.36 | 9.55E-07 |
| *HYAL1* | 0.36 | 1.40E-26 |
| *CTSW* | 0.36 | 2.26E-46 |
| *AP1M1* | 0.37 | 5.96E-04 |
| *PLA2G15* | 0.37 | 9.21E-08 |
| *ATP6AP1* | 0.37 | 4.13E-01 |
| *SGSH* | 0.38 | 9.55E-38 |
| *ACP5* | 0.39 | 1.35E-01 |
| *GUSB* | 0.39 | 5.83E-39 |
| *ACP2* | 0.39 | 4.99E-01 |
| *MAN2B1* | 0.40 | 5.12E-04 |
| *CTSG* | 0.42 | 2.71E-01 |
| *CTSV* | 0.43 | 2.32E-14 |
| *AP3D1* | 0.44 | 1.17E-01 |
| *GBA* | 0.45 | 1.72E-01 |
| *GGA1* | 0.48 | 3.34E-05 |
| *NPC2* | 0.48 | 6.32E-07 |
| *NAGA* | 0.50 | 2.57E-01 |
| *ATP6V0D1* | 0.50 | 3.33E-18 |
| *TPP1* | 0.51 | 6.40E-58 |
| *PSAP* | 0.52 | 1.57E-03 |
| *FUCA1* | 0.54 | 9.07E-28 |
| *GGA3* | 0.54 | 3.98E-03 |
| *DNASE2* | 0.54 | 2.26E-01 |
| *CD63* | 0.55 | 4.80E-03 |
| *LGMN* | 0.58 | 2.91E-23 |
| *GALNS* | 0.58 | 3.11E-05 |
| *LAPTM5* | 0.58 | 4.49E-01 |
| *CTSB* | 0.60 | 9.72E-48 |
| *AP4M1* | 0.62 | 1.08E-05 |
| *GLB1* | 0.62 | 2.26E-13 |
| *LAMP3* | 0.62 | 3.90E-03 |
| *ABCB9* | 0.62 | 2.16E-06 |
| *HGSNAT* | 0.65 | 6.00E-01 |
| *ATP6V0B* | 0.67 | 6.60E-02 |
| *PPT2* | 0.68 | 2.55E-01 |
| *NEU1* | 0.68 | 3.93E-01 |
| *ATP6V0D2* | 0.68 | 1.19E-03 |
| *AP1S1* | 0.70 | 1.08E-06 |
| *AGA* | 0.71 | 2.17E-23 |
| *LAPTM4A* | 0.71 | 6.18E-14 |
| *CD68* | 0.74 | 6.74E-01 |
| *CTSL* | 0.75 | 1.29E-04 |
| *CLTA* | 0.75 | 6.82E-07 |
| *M6PR* | 0.75 | 4.97E-37 |
| *SUMF1* | 0.77 | 1.35E-03 |
| *SLC11A1* | 0.77 | 6.10E-08 |
| *CTNS* | 0.78 | 6.20E-16 |
| *ATP6V0A4* | 0.79 | 2.51E-18 |
| *ATP6V0C* | 0.81 | 1.93E-01 |
| *GM2A* | 0.83 | 1.09E-18 |
| *ATP6V0A1* | 0.87 | 5.57E-01 |
| *AP1S3* | 0.88 | 2.26E-07 |
| *DNASE2B* | 0.88 | 1.04E-01 |
| *GNS* | 0.89 | 1.90E-11 |
| *ATP6V1H* | 0.89 | 1.82E-27 |
| *HEXB* | 0.90 | 2.28E-01 |
| *ASAH1* | 0.90 | 1.75E-21 |
| *GGA2* | 0.90 | 6.25E-06 |
| *GLA* | 0.93 | 2.59E-01 |
| *PPT1* | 1.02 | 5.31E-04 |
| *SLC17A5* | 1.02 | 5.53E-05 |
| *IGF2R* | 1.03 | 3.00E-03 |
| *LAPTM4B* | 1.07 | 4.57E-22 |
| *AP3S2* | 1.09 | 7.55E-01 |
| *CTSK* | 1.10 | 3.88E-34 |
| *CTSC* | 1.10 | 4.35E-03 |
| *NPC1* | 1.11 | 3.54E-02 |
| *CTSS* | 1.12 | 1.07E-05 |
| *AP3M1* | 1.13 | 6.94E-01 |
| *AP4B1* | 1.13 | 3.93E-04 |
| *CTSO* | 1.15 | 9.34E-07 |
| *LIPA* | 1.17 | 2.32E-01 |
| *CLTCL1* | 1.20 | 4.11E-03 |
| *AP3M2* | 1.24 | 2.45E-28 |
| *CLTC* | 1.24 | 9.16E-03 |
| *SCARB2* | 1.24 | 1.48E-42 |
| *SORT1* | 1.28 | 2.67E-42 |
| *MANBA* | 1.30 | 1.21E-20 |
| *LAMP2* | 1.32 | 2.16E-39 |
| *ARSB* | 1.38 | 5.09E-40 |
| *AP3B1* | 1.38 | 1.93E-05 |
| *AP3S1* | 1.39 | 1.95E-03 |
| *GALC* | 1.40 | 3.58E-01 |
| *ENTPD4* | 1.45 | 9.31E-05 |
| *CD164* | 1.46 | 1.24E-27 |
| *SLC11A2* | 1.52 | 8.73E-01 |
| *HEXA* | 1.54 | 1.00E-02 |
| *IDS* | 1.54 | 4.55E-12 |
| *AP1G1* | 1.60 | 5.16E-02 |
| *GNPTAB* | 1.76 | 8.25E-03 |
| *ARSG* | 1.79 | 1.04E-28 |
| *AP1S2* | 2.13 | 1.51E-01 |
| *AP4E1* | 2.28 | 2.67E-05 |
| *PSAPL1* | 2.37 | 8.40E-01 |
| *ATP6V0A2* | 2.47 | 1.79E-28 |
| *MFSD8* | 2.53 | 2.04E-02 |
| *AP4S1* | 3.08 | 5.10E-03 |
| *CLN3* | 3.26 | 2.14E-38 |
| *CLN5* | 4.37 | 2.02E-15 |

**Supplementary Table S2**. The expression changes of lysosomal genes (KEGG-lysosome) in *MEN1*-KD compared to Control from RNA-seq data.

| Gene | Fold Change | p-adj |
| --- | --- | --- |
| *ATP6V0D2* | 0.02 | 8.56E-12 |
| *LAMP3* | 0.13 | 1.39E-08 |
| *LAPTM5* | 0.30 | 4.99E-02 |
| *NPC1* | 0.32 | 5.05E-89 |
| *TPP1* | 0.35 | 8.76E-38 |
| *ATP6V0A4* | 0.41 | 8.82E-02 |
| *MCOLN1* | 0.41 | 2.69E-20 |
| *AP1S2* | 0.44 | 1.68E-12 |
| *CTNS* | 0.47 | 1.38E-19 |
| *CD164* | 0.47 | 3.31E-38 |
| *CTSV* | 0.49 | 8.56E-03 |
| *AP3B2* | 0.50 | 1.72E-01 |
| *AP3M2* | 0.51 | 2.77E-08 |
| *GALC* | 0.51 | 3.25E-09 |
| *ATP6V0A2* | 0.54 | 5.02E-13 |
| *IGF2R* | 0.55 | 1.50E-29 |
| *ARSG* | 0.56 | 2.49E-03 |
| *CTSA* | 0.56 | 9.50E-29 |
| *CLTCL1* | 0.60 | 1.15E-04 |
| *CLTC* | 0.60 | 3.72E-17 |
| *CTSB* | 0.64 | 1.39E-28 |
| *DNASE2* | 0.65 | 2.81E-09 |
| *GUSB* | 0.66 | 2.33E-06 |
| *AP3D1* | 0.67 | 1.79E-20 |
| *CTSS* | 0.67 | 8.45E-13 |
| *TCIRG1* | 0.67 | 8.32E-12 |
| *PSAP* | 0.68 | 4.89E-28 |
| *AP3S2* | 0.68 | 2.29E-06 |
| *AP4B1* | 0.69 | 7.45E-03 |
| *ARSA* | 0.69 | 1.16E-03 |
| *NAGPA* | 0.70 | 6.77E-04 |
| *IDUA* | 0.70 | 2.71E-02 |
| *ACP2* | 0.70 | 5.93E-06 |
| *AP3S1* | 0.71 | 9.97E-08 |
| *AP4E1* | 0.72 | 4.73E-02 |
| *CTSF* | 0.72 | 2.52E-05 |
| *ARSB* | 0.75 | 1.27E-02 |
| *NEU1* | 0.76 | 1.57E-05 |
| *AP3B1* | 0.76 | 2.99E-03 |
| *NAGLU* | 0.77 | 2.95E-06 |
| *GAA* | 0.77 | 9.89E-06 |
| *ATP6V0A1* | 0.77 | 1.21E-04 |
| *CTSZ* | 0.78 | 1.29E-06 |
| *CTSD* | 0.79 | 4.96E-08 |
| *GLB1* | 0.79 | 8.40E-05 |
| *LAMP1* | 0.80 | 1.17E-06 |
| *PSAPL1* | 0.80 | 8.20E-01 |
| *LAMP2* | 0.81 | 4.47E-04 |
| *GALNS* | 0.82 | 9.73E-03 |
| *GLA* | 0.82 | 2.27E-05 |
| *GNS* | 0.82 | 1.07E-04 |
| *AP4M1* | 0.82 | 1.33E-01 |
| *CTSL* | 0.83 | 2.87E-06 |
| *HEXB* | 0.84 | 1.45E-03 |
| *ATP6V0B* | 0.85 | 1.75E-03 |
| *LGMN* | 0.85 | 1.38E-02 |
| *CTSO* | 0.87 | 6.78E-01 |
| *AP1G1* | 0.87 | 8.30E-02 |
| *MANBA* | 0.93 | 7.52E-01 |
| *SGSH* | 0.94 | 5.47E-01 |
| *ACP5* | 0.95 | 9.58E-01 |
| *CD63* | 0.95 | 2.71E-01 |
| *AP3M1* | 0.96 | 7.19E-01 |
| *CTSC* | 0.98 | 8.11E-01 |
| *SCARB2* | 0.99 | 9.52E-01 |
| *PPT1* | 1.00 | 9.79E-01 |
| *CTSK* | 1.05 | 9.53E-01 |
| *HEXA* | 1.05 | 5.31E-01 |
| *ATP6AP1* | 1.06 | 2.57E-01 |
| *GNPTAB* | 1.06 | 7.59E-01 |
| *IDS* | 1.09 | 3.10E-01 |
| *CLTA* | 1.10 | 4.28E-02 |
| *GGA3* | 1.10 | 2.47E-01 |
| *SLC11A2* | 1.11 | 1.42E-01 |
| *LAPTM4A* | 1.15 | 7.38E-04 |
| *GGA1* | 1.16 | 9.94E-02 |
| *MAN2B1* | 1.16 | 1.94E-02 |
| *HGSNAT* | 1.20 | 2.33E-02 |
| *FUCA1* | 1.23 | 5.36E-02 |
| *AGA* | 1.24 | 2.90E-02 |
| *SLC17A5* | 1.27 | 1.01E-02 |
| *AP1B1* | 1.30 | 4.90E-07 |
| *M6PR* | 1.31 | 1.85E-09 |
| *ATP6V0D1* | 1.34 | 1.41E-09 |
| *ATP6V0C* | 1.35 | 2.71E-02 |
| *ABCA2* | 1.35 | 8.26E-06 |
| *ASAH1* | 1.36 | 6.43E-09 |
| *NAGA* | 1.36 | 3.66E-02 |
| *SLC11A1* | 1.38 | 6.10E-01 |
| *LAPTM4B* | 1.39 | 1.05E-11 |
| *CLN5* | 1.39 | 3.62E-01 |
| *ATP6V1H* | 1.39 | 3.89E-08 |
| *NPC2* | 1.40 | 3.39E-12 |
| *GNPTG* | 1.40 | 1.40E-06 |
| *AP1M1* | 1.41 | 6.63E-09 |
| *CTSH* | 1.42 | 8.35E-06 |
| *CLTB* | 1.52 | 3.49E-16 |
| *GGA2* | 1.54 | 1.80E-15 |
| *MFSD8* | 1.57 | 4.71E-03 |
| *AP4S1* | 1.60 | 3.61E-05 |
| *SORT1* | 1.61 | 7.02E-07 |
| *SUMF1* | 1.65 | 2.16E-11 |
| *GM2A* | 1.78 | 8.02E-20 |
| *ENTPD4* | 1.87 | 2.06E-12 |
| *LIPA* | 2.04 | 7.33E-63 |
| *AP1S1* | 2.05 | 1.50E-52 |
| *AP1M2* | 2.06 | 3.56E-18 |
| *PPT2* | 2.15 | 1.28E-03 |
| *SMPD1* | 2.19 | 2.57E-12 |
| *PLA2G15* | 2.27 | 2.81E-12 |
| *ABCB9* | 2.37 | 4.23E-13 |
| *AP1S3* | 2.67 | 2.11E-32 |
| *NAPSA* | 2.92 | 6.57E-02 |
| *HYAL1* | 4.61 | 1.13E-05 |
| *CLN3* | 8.95 | 4.37E-02 |

**Supplementary Table S3**. The expression changes of autophagic genes (Reactome-Autophagy) in *MEN1*-KD compared to Control from RNA-seq data.

| Gene | Fold Change | p-adj |
| --- | --- | --- |
| *MAP1LC3B* | 0.18 | 0.00E+00 |
| *SQSTM1* | 0.22 | 0.00E+00 |
| *WIPI1* | 0.23 | 6.20E-88 |
| *HSP90AA1* | 0.32 | 0.00E+00 |
| *VIM* | 0.40 | 3.25E-124 |
| *PLIN2* | 0.41 | 8.07E-58 |
| *TOMM5* | 0.45 | 1.73E-01 |
| *GFAP* | 0.47 | 4.00E-02 |
| *LAMTOR5* | 0.48 | 6.06E-25 |
| *UBC* | 0.50 | 6.51E-75 |
| *DYNC1H1* | 0.51 | 4.63E-51 |
| *WDR45* | 0.51 | 5.27E-18 |
| *UVRAG* | 0.53 | 1.50E-07 |
| *HSP90AB1* | 0.55 | 1.91E-77 |
| *ATG13* | 0.55 | 3.47E-42 |
| *TSC1* | 0.56 | 1.06E-07 |
| *RB1CC1* | 0.56 | 1.11E-03 |
| *IFT88* | 0.57 | 3.41E-02 |
| *ARL13B* | 0.60 | 5.74E-03 |
| *TUBB2B* | 0.63 | 1.80E-04 |
| *MLST8* | 0.63 | 5.35E-18 |
| *ATG101* | 0.64 | 4.72E-13 |
| *ATG5* | 0.64 | 1.28E-04 |
| *HDAC6* | 0.65 | 4.53E-08 |
| *ULK1* | 0.66 | 3.27E-12 |
| *VPS37C* | 0.67 | 3.00E-07 |
| *PRKAB2* | 0.68 | 2.07E-03 |
| *MTMR14* | 0.68 | 1.47E-11 |
| *PRKAG2* | 0.69 | 1.08E-08 |
| *PRKAG1* | 0.69 | 5.77E-01 |
| *CHMP4C* | 0.69 | 5.17E-03 |
| *ATG14* | 0.70 | 3.49E-04 |
| *MFN1* | 0.72 | 1.01E-02 |
| *ATG7* | 0.73 | 3.53E-05 |
| *CSNK2A2* | 0.73 | 1.87E-05 |
| *UBB* | 0.74 | 1.27E-09 |
| *USP30* | 0.75 | 3.47E-02 |
| *MTOR* | 0.76 | 2.28E-05 |
| *ATM* | 0.76 | 2.10E-01 |
| *EEF1A1* | 0.77 | 4.86E-14 |
| *VPS28* | 0.77 | 7.66E-07 |
| *SLC38A9* | 0.77 | 8.48E-03 |
| *VCP* | 0.78 | 6.59E-13 |
| *TUBA1C* | 0.80 | 3.23E-07 |
| *LAMP2* | 0.81 | 4.47E-04 |
| *PRKAA1* | 0.81 | 6.58E-02 |
| *HSF1* | 0.83 | 3.21E-04 |
| *VPS37B* | 0.84 | 1.59E-02 |
| *CSNK2A1* | 0.84 | 7.39E-05 |
| *GABARAPL1* | 0.86 | 9.01E-03 |
| *ATG3* | 0.88 | 1.44E-01 |
| *AMBRA1* | 0.88 | 8.65E-02 |
| *TUBB2A* | 0.88 | 1.75E-01 |
| *HSPA8* | 0.90 | 5.10E-03 |
| *ATG4B* | 0.90 | 1.05E-01 |
| *PEX5* | 0.90 | 1.99E-01 |
| *PIK3C3* | 0.91 | 5.00E-01 |
| *DYNC1I1* | 0.92 | 9.57E-01 |
| *MVB12A* | 0.93 | 3.69E-01 |
| *CSNK2B* | 0.93 | 6.32E-01 |
| *TSG101* | 0.93 | 2.25E-01 |
| *RRAGC* | 0.96 | 7.18E-01 |
| *TOMM20* | 0.96 | 3.89E-01 |
| *CHMP3* | 0.97 | 6.85E-01 |
| *PIK3R4* | 0.97 | 8.92E-01 |
| *TUBB6* | 0.98 | 7.83E-01 |
| *VPS37A* | 0.98 | 8.71E-01 |
| *MTMR3* | 0.99 | 9.65E-01 |
| *RRAGA* | 1.00 | 9.63E-01 |
| *VDAC1* | 1.00 | 9.61E-01 |
| *ATG9A* | 1.00 | 9.89E-01 |
| *NBR1* | 1.01 | 8.59E-01 |
| *CHMP6* | 1.02 | 8.92E-01 |
| *CHMP4A* | 1.02 | 9.74E-01 |
| *VPS37D* | 1.03 | 9.05E-01 |
| *MVB12B* | 1.04 | 8.08E-01 |
| *TOMM40* | 1.05 | 3.85E-01 |
| *RHEB* | 1.06 | 2.78E-01 |
| *DYNLL2* | 1.06 | 4.32E-01 |
| *UBAP1* | 1.07 | 2.94E-01 |
| *TUBB4B* | 1.07 | 2.43E-01 |
| *ATG4C* | 1.07 | 8.23E-01 |
| *ATG4D* | 1.07 | 5.56E-01 |
| *DYNC1LI1* | 1.08 | 2.87E-01 |
| *GABARAP* | 1.08 | 7.96E-01 |
| *PCNT* | 1.08 | 6.57E-01 |
| *TOMM22* | 1.08 | 3.10E-01 |
| *PRKAB1* | 1.10 | 1.88E-01 |
| *BECN1* | 1.11 | 1.28E-01 |
| *DYNC1I2* | 1.12 | 1.38E-01 |
| *WDR45B* | 1.12 | 2.05E-02 |
| *TUBA1B* | 1.13 | 1.14E-02 |
| *DYNC1LI2* | 1.13 | 1.75E-01 |
| *PARK7* | 1.14 | 5.17E-03 |
| *GABARAPL2* | 1.15 | 3.45E-02 |
| *TUBA4A* | 1.16 | 1.48E-02 |
| *CHMP2A* | 1.17 | 7.77E-04 |
| *CHMP7* | 1.18 | 9.92E-03 |
| *LAMTOR1* | 1.18 | 6.37E-03 |
| *CHMP2B* | 1.19 | 9.61E-03 |
| *ATG9B* | 1.20 | 9.40E-01 |
| *UBA52* | 1.22 | 7.06E-05 |
| *ATG12* | 1.22 | 1.69E-03 |
| *TSC2* | 1.23 | 1.02E-03 |
| *UBE2N* | 1.23 | 8.13E-05 |
| *PINK1* | 1.25 | 1.90E-02 |
| *MFN2* | 1.25 | 7.09E-06 |
| *LAMTOR4* | 1.26 | 8.25E-03 |
| *RPS27A* | 1.26 | 3.13E-09 |
| *PGAM5* | 1.27 | 1.48E-04 |
| *LAMTOR3* | 1.27 | 9.14E-03 |
| *TOMM70* | 1.28 | 6.97E-06 |
| *ATG16L1* | 1.28 | 4.59E-05 |
| *TOMM7* | 1.29 | 6.61E-05 |
| *MAP1LC3A* | 1.31 | 1.26E-01 |
| *RPTOR* | 1.34 | 7.37E-06 |
| *TUBAL3* | 1.35 | 5.60E-01 |
| *EPAS1* | 1.39 | 1.93E-19 |
| *PRKAA2* | 1.40 | 1.08E-01 |
| *CHMP4B* | 1.42 | 6.27E-16 |
| *TUBB3* | 1.47 | 7.90E-02 |
| *LAMTOR2* | 1.47 | 7.67E-06 |
| *ATG10* | 1.50 | 2.33E-04 |
| *WIPI2* | 1.51 | 1.66E-10 |
| *MTERF3* | 1.53 | 9.31E-04 |
| *RRAGD* | 1.56 | 1.28E-05 |
| *PLIN3* | 1.56 | 7.78E-17 |
| *UBE2V1* | 1.58 | 1.08E-01 |
| *SRC* | 1.59 | 5.57E-31 |
| *ATG4A* | 1.64 | 2.11E-05 |
| *RRAGB* | 1.67 | 3.84E-05 |
| *DYNLL1* | 1.69 | 2.00E-37 |
| *TUBA3D* | 1.77 | 4.14E-01 |
| *TUBA1A* | 1.79 | 2.21E-30 |
| *FUNDC1* | 1.90 | 1.13E-12 |
| *TUBA4B* | 2.49 | 4.88E-01 |
| *MAP1LC3C* | 2.97 | 6.15E-02 |
| *TUBB4A* | 3.82 | 4.29E-23 |

**Supplementary Table S4**. The expression changes of mitochondrial genes (Wong-Mitochondria-Gene-Module) in *MEN1*-KD compared to Control from RNA-seq data.

| Gene | Fold Change | p-adj |
| --- | --- | --- |
| *PCK2* | 0.14 | 0.00E+00 |
| *MTHFD2* | 0.20 | 0.00E+00 |
| *GRPEL2* | 0.31 | 1.92E-22 |
| *WARS2* | 0.32 | 3.06E-12 |
| *TSFM* | 0.36 | 9.03E-02 |
| *SLC25A32* | 0.37 | 7.73E-02 |
| *TFAM* | 0.38 | 4.16E-29 |
| *BCL2* | 0.39 | 6.92E-02 |
| *SFXN2* | 0.50 | 6.13E-05 |
| *GCDH* | 0.53 | 1.37E-09 |
| *LONP1* | 0.59 | 7.05E-33 |
| *FIBP* | 0.59 | 1.01E-18 |
| *SLC16A5* | 0.60 | 1.72E-13 |
| *PDP1* | 0.61 | 1.52E-06 |
| *ATP13A3* | 0.63 | 9.34E-05 |
| *MRPS16* | 0.64 | 2.18E-29 |
| *DBT* | 0.66 | 9.62E-02 |
| *FH* | 0.67 | 1.26E-13 |
| *HAX1* | 0.68 | 1.25E-11 |
| *NQO1* | 0.69 | 1.54E-18 |
| *NDUFA7* | 0.69 | 6.25E-01 |
| *MRPL18* | 0.70 | 2.05E-14 |
| *LRPPRC* | 0.73 | 8.28E-06 |
| *MRPL49* | 0.73 | 1.74E-07 |
| *SDHB* | 0.74 | 3.31E-09 |
| *MRPS18B* | 0.75 | 1.88E-07 |
| *GPX4* | 0.76 | 1.31E-08 |
| *ATP5F1E* | 0.76 | 5.44E-02 |
| *GSTZ1* | 0.76 | 3.19E-04 |
| *MRPL3* | 0.76 | 1.52E-07 |
| *AGMAT* | 0.77 | 2.72E-02 |
| *CPT1A* | 0.78 | 9.67E-06 |
| *DUT* | 0.78 | 2.00E-04 |
| *GOT2* | 0.78 | 1.08E-09 |
| *ALDH6A1* | 0.79 | 1.28E-01 |
| *NDUFA9* | 0.80 | 1.75E-02 |
| *DAP3* | 0.80 | 4.70E-05 |
| *COX8A* | 0.81 | 8.85E-05 |
| *ATP6V1C1* | 0.82 | 1.68E-03 |
| *SLC25A39* | 0.82 | 1.46E-05 |
| *COX7A1* | 0.82 | 8.85E-01 |
| *MRPS18A* | 0.83 | 3.82E-02 |
| *COX7C* | 0.83 | 1.38E-03 |
| *AK2* | 0.83 | 7.36E-05 |
| *SLC25A22* | 0.83 | 3.30E-02 |
| *MTRR* | 0.84 | 3.69E-02 |
| *ATP1B3* | 0.84 | 7.44E-04 |
| *MRPL45* | 0.85 | 6.31E-03 |
| *CYC1* | 0.86 | 6.29E-03 |
| *PDHX* | 0.86 | 2.47E-01 |
| *COX11* | 0.86 | 2.06E-02 |
| *ABCB6* | 0.87 | 6.88E-01 |
| *MIPEP* | 0.87 | 1.27E-01 |
| *SURF1* | 0.88 | 1.74E-01 |
| *NDUFS3* | 0.88 | 4.59E-02 |
| *NT5M* | 0.88 | 5.41E-01 |
| *ATP5MC1* | 0.89 | 4.93E-02 |
| *ATP6V1D* | 0.89 | 9.36E-02 |
| *MRPS18C* | 0.90 | 2.59E-01 |
| *SEC61G* | 0.90 | 5.41E-01 |
| *COX6A1* | 0.90 | 1.34E-01 |
| *CLPP* | 0.92 | 2.02E-01 |
| *SLC25A19* | 0.92 | 5.68E-01 |
| *COX7A2L* | 0.92 | 7.57E-02 |
| *NDUFB9* | 0.93 | 2.14E-01 |
| *COX7A2* | 0.93 | 3.57E-01 |
| *CYP27A1* | 0.93 | 9.69E-01 |
| *HSCB* | 0.95 | 7.15E-01 |
| *MCCC2* | 0.95 | 5.03E-01 |
| *ECI1* | 0.95 | 4.72E-01 |
| *MRPS30* | 0.95 | 5.10E-01 |
| *BOP1* | 0.95 | 3.38E-01 |
| *VDAC2* | 0.96 | 3.72E-01 |
| *TIMM13* | 0.96 | 6.17E-01 |
| *COX6B1* | 0.97 | 6.05E-01 |
| *ATP5F1B* | 0.97 | 5.59E-01 |
| *NIPSNAP1* | 0.97 | 6.78E-01 |
| *MRPL11* | 0.98 | 7.47E-01 |
| *TIMM17B* | 0.99 | 8.56E-01 |
| *MRPL32* | 1.00 | 9.49E-01 |
| *TUFM* | 1.00 | 9.78E-01 |
| *ATP5MC2* | 1.01 | 9.18E-01 |
| *COX5A* | 1.01 | 9.23E-01 |
| *NDUFS8* | 1.01 | 9.02E-01 |
| *COX4I1* | 1.01 | 8.43E-01 |
| *TRIM45* | 1.01 | 9.69E-01 |
| *CP* | 1.02 | 9.13E-01 |
| *NDUFV1* | 1.02 | 7.72E-01 |
| *UNG* | 1.02 | 7.66E-01 |
| *ATP8A1* | 1.03 | 9.86E-01 |
| *TIMM10* | 1.03 | 6.88E-01 |
| *NDUFB10* | 1.03 | 6.52E-01 |
| *MRPS15* | 1.04 | 5.64E-01 |
| *MRPL42* | 1.04 | 6.09E-01 |
| *TOMM40* | 1.05 | 3.85E-01 |
| *HINT2* | 1.05 | 9.52E-01 |
| *HCCS* | 1.05 | 5.16E-01 |
| *MRPL12* | 1.05 | 5.52E-01 |
| *KARS1* | 1.06 | 2.63E-01 |
| *ATP6AP1* | 1.06 | 2.57E-01 |
| *SUCLG1* | 1.06 | 4.08E-01 |
| *SOD2* | 1.06 | 1.44E-01 |
| *NDUFV2* | 1.07 | 8.99E-01 |
| *CRAT* | 1.07 | 3.55E-01 |
| *TSPO* | 1.07 | 3.16E-01 |
| *GLUD1* | 1.07 | 1.86E-01 |
| *COX17* | 1.08 | 3.82E-01 |
| *NDUFB1* | 1.08 | 6.07E-01 |
| *NDUFA3* | 1.08 | 5.06E-01 |
| *NDUFB2* | 1.08 | 1.55E-01 |
| *GSR* | 1.09 | 3.96E-02 |
| *ECHS1* | 1.09 | 5.58E-02 |
| *DECR1* | 1.09 | 3.20E-01 |
| *PDHA1* | 1.11 | 1.03E-01 |
| *NDUFS6* | 1.11 | 2.01E-01 |
| *CKMT1B* | 1.12 | 7.82E-01 |
| *MRPL27* | 1.12 | 8.75E-02 |
| *NDUFB8* | 1.12 | 2.67E-01 |
| *ATP6V1F* | 1.13 | 2.77E-02 |
| *ATP5PD* | 1.14 | 1.49E-02 |
| *ATP5ME* | 1.14 | 5.95E-01 |
| *NDUFA1* | 1.15 | 3.94E-01 |
| *FDXR* | 1.16 | 6.34E-03 |
| *MRPL13* | 1.17 | 2.12E-02 |
| *PPIF* | 1.17 | 4.67E-04 |
| *NDUFAB1* | 1.18 | 1.17E-02 |
| *CRYZ* | 1.18 | 1.84E-02 |
| *SCO1* | 1.18 | 5.12E-03 |
| *ATP5PO* | 1.18 | 7.35E-02 |
| *NDUFS4* | 1.18 | 6.43E-03 |
| *NDUFS2* | 1.19 | 3.91E-03 |
| *MRPS36* | 1.19 | 3.32E-02 |
| *NDUFC1* | 1.20 | 1.13E-01 |
| *COQ3* | 1.22 | 2.15E-01 |
| *NDUFA10* | 1.23 | 3.56E-05 |
| *UQCRFS1* | 1.24 | 1.42E-05 |
| *MTX2* | 1.24 | 1.06E-03 |
| *AIFM1* | 1.24 | 2.13E-05 |
| *ATP5F1C* | 1.25 | 3.19E-08 |
| *NDUFA6* | 1.25 | 2.99E-04 |
| *UQCR11* | 1.26 | 1.57E-02 |
| *TIMM23* | 1.27 | 7.22E-07 |
| *NDUFB4* | 1.27 | 2.23E-05 |
| *ACADSB* | 1.27 | 7.65E-02 |
| *MRPS33* | 1.27 | 5.57E-05 |
| *HSD17B10* | 1.27 | 3.66E-07 |
| *TOMM70* | 1.28 | 6.97E-06 |
| *TOMM7* | 1.29 | 6.61E-05 |
| *GCAT* | 1.29 | 1.57E-01 |
| *COX7B* | 1.29 | 9.49E-02 |
| *GATD3* | 1.30 | 3.86E-02 |
| *CRYZL1* | 1.31 | 4.10E-02 |
| *ACAT1* | 1.31 | 1.83E-04 |
| *COX5B* | 1.31 | 2.02E-06 |
| *MRPS17* | 1.32 | 9.09E-02 |
| *MRPL40* | 1.32 | 7.18E-04 |
| *ATP5MF* | 1.32 | 3.87E-06 |
| *MCEE* | 1.33 | 1.53E-01 |
| *ATP5PB* | 1.33 | 1.03E-09 |
| *NDUFB6* | 1.34 | 1.11E-06 |
| *GPD2* | 1.34 | 3.52E-03 |
| *PCCB* | 1.37 | 4.51E-07 |
| *ATP6V1E1* | 1.37 | 2.70E-11 |
| *NDUFA8* | 1.38 | 1.88E-07 |
| *PRDX5* | 1.40 | 3.72E-11 |
| *NDUFB5* | 1.41 | 1.48E-03 |
| *UQCRQ* | 1.41 | 2.68E-02 |
| *COX6C* | 1.42 | 2.53E-13 |
| *ATP5PF* | 1.42 | 2.67E-08 |
| *NME4* | 1.44 | 4.39E-13 |
| *MRPS28* | 1.46 | 1.04E-03 |
| *NDUFAF1* | 1.46 | 4.80E-04 |
| *SLC9A2* | 1.46 | 1.94E-01 |
| *NDUFA4* | 1.47 | 1.72E-17 |
| *GSTO1* | 1.47 | 7.89E-16 |
| *UQCRB* | 1.48 | 1.81E-12 |
| *MCAT* | 1.48 | 4.34E-07 |
| *NDUFA12* | 1.50 | 1.71E-08 |
| *MRPL36* | 1.52 | 5.83E-10 |
| *VDAC3* | 1.53 | 1.09E-20 |
| *IDH2* | 1.53 | 4.79E-10 |
| *SLC9A5* | 1.53 | 7.16E-02 |
| *NDUFB3* | 1.54 | 1.30E-09 |
| *SFXN5* | 1.54 | 1.63E-07 |
| *SLC25A17* | 1.57 | 6.39E-09 |
| *UQCR10* | 1.57 | 8.66E-12 |
| *SUCLA2* | 1.58 | 1.12E-06 |
| *SCP2* | 1.59 | 1.06E-05 |
| *DCTN6* | 1.59 | 6.21E-09 |
| *SLC25A1* | 1.60 | 4.76E-13 |
| *PDK3* | 1.61 | 1.68E-05 |
| *ATP5MG* | 1.62 | 1.95E-12 |
| *ATP6V1G1* | 1.63 | 1.74E-20 |
| *DLD* | 1.63 | 2.26E-15 |
| *ATOX1* | 1.63 | 1.04E-08 |
| *AUH* | 1.64 | 8.08E-04 |
| *NDUFC2* | 1.65 | 4.75E-09 |
| *ATP5MC3* | 1.66 | 2.81E-29 |
| *ATP1B1* | 1.67 | 3.66E-27 |
| *SLC16A3* | 1.67 | 3.04E-17 |
| *NDUFA5* | 1.68 | 2.49E-10 |
| *COX18* | 1.74 | 4.52E-10 |
| *NDUFS5* | 1.76 | 2.74E-37 |
| *CYB5A* | 1.77 | 5.44E-24 |
| *PTRH2* | 1.79 | 2.62E-24 |
| *TIMM8B* | 1.85 | 2.74E-26 |
| *OXCT1* | 1.85 | 2.53E-03 |
| *DIABLO* | 1.99 | 4.76E-03 |
| *CHDH* | 2.11 | 5.53E-06 |
| *PDHB* | 2.19 | 3.98E-57 |
| *MAOA* | 2.27 | 4.18E-06 |
| *NFS1* | 2.28 | 1.46E-20 |
| *SCO2* | 2.54 | 1.47E-01 |
| *SLC40A1* | 9.68 | 1.52E-29 |

**Supplementary Table S5**. Nucleotide sequences.

| Used for | Name | Sequence: 5' to 3' |
| --- | --- | --- |
| Genotyping | *Men1*-flox-P1 | TCCAGTCCCTCTTCAGCTTC |
| Genotyping | *Men1*-flox-P2 | GCCATTTCATTACCTCTTTCTCCG |
| Genotyping | *Men1*-flox-P3 | TACCACTGCAAAGGCCACGC |
| Genotyping | *Men1*-KO-P1 | CCCACATCCAGTCCCTCTTCAGCT |
| Genotyping | *Men1*-KO-P2 | AAGGTACAGCAGAGGTCACAGAG |
| Genotyping | *Men1*-KO-P3 | GACAGGATTGGGAATTCTCTTTT |
| Genotyping | LSL-*Kras^G12D^*-P1 | GTCTTTCCCCAGCACAGTGC |
| Genotyping | LSL-*Kras^G12D^*-P2 | CTCTTGCCTACGCCACCAGCTC |
| Genotyping | LSL-*Kras^G12D^*-P3 | AGCTAGCCACCATGGCTTGAGTAAGTCTGCA |
| Genotyping | *Ubc-Cre*-F | GCGGTCTGGCAGTAAAAACTATC |
| Genotyping | *Ubc-Cre*-R | GTGAAACAGCATTGCTGTCACTT |
| Genotyping | *Mll1*-flox-P1 | TCTCTGAAGTAAGCCTTTCTTAG |
| Genotyping | *Mll1*-flox-P2 | CAGTGGACATTCCAACTCTTCAA |
| Genotyping | *Mll1*-flox-P3 | CACCCAGCATTGCAGAGTCAG |
| Genotyping | *Sftpc-Cre*-P1 | ACACCGGCCTTATTCCAA G |
| Genotyping | *Sftpc-Cre*-P2 | TGCTTCACAGGGTCGGTAG |
| Genotyping | *Sftpc-Cre*-P3 | CAT TAC CTG GGG TAG GAC CA |
| Plasmids construction | PLNCX2-*TFE3*-F | AAATATATAAGCTTATGTCTCATGCGGCCGAACC |
| Plasmids construction | PLNCX2-*TFE3*-R | AAATATATGTCGACTCAAGCGTAGTCTGGGACGTCGTATGGGTAGGACTCCTCTTCCATGCTGA |
| Plasmids construction | PLNCX2-*TFEB*-F | AAATATATAAGCTTATGGCGTCACGCATAGGGTT |
| Plasmids construction | PLNCX2-*TFEB*-R | AAATATATGTCGACTCAAGCGTAGTCTGGGACGTCGTATGGGTACAGCACATCGCCCTCCTCCA |
| Plasmids construction | PLNCX2-*MEN1*-F | AAATATATAAGCTTATGGGGCTGAAGGCCGCCCA |
| Plasmids construction | PLNCX2-*MEN1*-R | AAATATATGTCGACTCACTTGTCATCGTCGTCCTTGTAATCGAGGCCTTTGCGCTGCCGCT |
| RT-qPCR | Homo sapiens-*ACTB*-F | TCAGAAGGATTCCTATGTGGGCGA |
| RT-qPCR | Homo sapiens-*ACTB*-R | TTTCTCCATGTCGTCCCAGTTGGT |
| RT-qPCR | Homo sapiens-*MEN1*-F | ATCACAGGCACCAAATTGGACAGC |
| RT-qPCR | Homo sapiens-*MEN1*-R | AACACTACCCAGGCATGATCCTCA |
| RT-qPCR | Homo sapiens-*ATP6V0C*-F | CCTCATCCTCTCCACAAAGTAG |
| RT-qPCR | Homo sapiens- *ATP6V0C*-R | CGCATGTACAAGACCAACTAC |
| RT-qPCR | Homo sapiens-*CTSD*-F | AACTGCTGGACATCGCTTGCT |
| RT-qPCR | Homo sapiens- *CTSD*-R | CATTCTTCACGTAGGTGCTGGA |
| RT-qPCR | Homo sapiens-*GAA*-F | CGCTGATTGGGAAGGTATGG |
| RT-qPCR | Homo sapiens-*GAA*-R | AGTTGGAAGGCTCGTTCATG |
| RT-qPCR | Homo sapiens-*TCIRG1*-F | ACACTCATCCGCACCAAC |
| RT-qPCR | Homo sapiens-*TCIRG1*-R | CCGAACATCACAGCAAACAG |
| RT-qPCR | Homo sapiens-*NAPSA*-F | GTAGATGGAATCCTGAGCGAG |
| RT-qPCR | Homo sapiens-*NAPSA*-R | AGACAGAATGGGAAAACCGAG |
| RT-qPCR | Homo sapiens-*CTSB*-F | CTTGAAGAGGCTATGTGGTACC |
| RT-qPCR | Homo sapiens-*CTSB*-R | CCCTGGTCTCTGATCTCTTTG |
| RT-qPCR | Homo sapiens-*CTSE*-F | CTTCCCAGTCCAGCACATAC |
| RT-qPCR | Homo sapiens-*CTSE*-R | GTGACACTTTCTCCAAACTGC |
| RT-qPCR | Homo sapiens-*MITF*-F | AGGAAATCTTGGGCTTGATGG |
| RT-qPCR | Homo sapiens-*MITF*-R | TGTTGGGAAGGTTGGCTG |
| RT-qPCR | Homo sapiens-*TFEB*-F | CCAGAAGCGAGAGCTCACAGAT |
| RT-qPCR | Homo sapiens-*TFEB*-R | TGTGATTGTCTTTCTTCTGCCG |
| RT-qPCR | Homo sapiens-*TFE3*-F | GCTGCTTTCCTTGGC |
| RT-qPCR | Homo sapiens-*TFE3*-R | ATCTGAGGGCGGTGC |
| RT-qPCR | Homo sapiens-*MAP1LC3B*-F | TATCACCGGGATTTTGGTTG |
| RT-qPCR | Homo sapiens-*MAP1LC3B*-R | GAGAAGACCTTCAAGCAGCG |
| RT-qPCR | Homo sapiens-*SQSTM1*-F | AATCAGCTTCTGGTCCATCG |
| RT-qPCR | Homo sapiens-*SQSTM1*-R | TTCTTTTCCCTCCGTGCTC |
| RT-qPCR | Homo sapiens-*ATG5*-F | AGCAACTCTGGATGGGATTG |
| RT-qPCR | Homo sapiens-*ATG5*-R | AGGTCTTTCAGTCGTTGTCTG |
| RT-qPCR | Homo sapiens-*ATG7*-F | TTTTGCTATCCTGCCCTCTG |
| RT-qPCR | Homo sapiens-*ATG7*-R | GCTGTGACTCCTTCTGTTTGAC |
| RT-qPCR | Homo sapiens-*MLL1*-F | ATCTCTGACCACACACCTTGGCTT |
| RT-qPCR | Homo sapiens-*MLL1*-R | TCCACCTAAATGTCGGTTCTCGCA |
| RT-qPCR | Homo sapiens-*TFAM*-F | ATGGCGTTTCTCCGAAGCAT |
| RT-qPCR | Homo sapiens-*TFAM*-R | TCCGCCCTATAAGCATCTTGA |
| RT-qPCR | Homo sapiens-*HB2M*-F | TGTTCCTGCTGGGTAGCTCT |
| RT-qPCR | Homo sapiens-*HB2M*-R | CCTCCATGATGCTGCTTACA |
| RT-qPCR | Homo sapiens-*HSPA1A*-F | CTTGGCCGAGAAGGACGAGT |
| RT-qPCR | Homo sapiens-*HSPA1A*-R | AACAGCAATCTTGGAAAGGCCC |
| RT-qPCR | Homo sapiens-*HSPA1B*-F | CCCCATCATCAGCGGACTGT |
| RT-qPCR | Homo sapiens-*HSPA1B*-R | GCAGCAAAGTCCTTGAGTCCC |
| RT-qPCR | Homo sapiens-*HSP90AA1*-F | TCAGTTGCTTCAGCGTCC |
| RT-qPCR | Homo sapiens-*HSP90AA1*-R | GGTCTTGGGTCTGGGTTTC |
| RT-qPCR | Homo sapiens-*DNAJB1*-F | CTGGCTCTGCAAAAGAATGTG |
| RT-qPCR | Homo sapiens-*DNAJB1*-R | CTGAACCATTCCAGGTCCTATC |
| RT-qPCR | Homo sapiens-*mtND1*-mtDNA-F | CACTTTCCACACAGACATCA |
| RT-qPCR | Homo sapiens- *mtND1*-mtDNA-R | TGGTTAGGCTGGTGTTAGGG |
| RT-qPCR | Homo sapiens-*mtND1*-F | CGACCTGACAGAAGGAGA |
| RT-qPCR | Homo sapiens-*mtND1*-R | GTAACGGAAGCGTGGATA |
| RT-qPCR | Homo sapiens-*mtND2*-F | CCTTGCCATCATCTACTTCA |
| RT-qPCR | Homo sapiens-*mtND2*-R | TTGAGGCTGTTGCTTGTGT |
| RT-qPCR | Mus musculus-*Atp6v0c*-F | GCCCTAATCCTCTCCACAAAG |
| RT-qPCR | Mus musculus-*Atp6v0c*-R | GGACACTGCACATTTACAAGAC |
| RT-qPCR | Mus musculus-*Ctsd*-F | TGACAAGTCCAGCACCTATG |
| RT-qPCR | Mus musculus-*Ctsd*-R | CTCCACCTTGATACCTCTTGC |
| RT-qPCR | Mus musculus-*Gaa*-F | ACAATGAACTGGAGAACCCC |
| RT-qPCR | Mus musculus-*Gaa*-R | GGTTGTGGAGGTTGTAGTGTG |
| RT-qPCR | Mus musculus-*Ctse*-F | AACCCAACTGACTACATCCTG |
| RT-qPCR | Mus musculus-*Ctse*-R | CAAAGACTGAGTAGAACTGTCGG |
| RT-qPCR | Mus musculus-*Tcirg1*-F | CTTCACCTCCAGCTTCCAG |
| RT-qPCR | Mus musculus-*Tcirg1*-R | CACATCGCCAAACATCACAG |
| RT-qPCR | Mus musculus-*Napsa*-F | TCTAGTCCCCAGAGATGTCG |
| RT-qPCR | Mus musculus-*Napsa*-R | AAGGTGGATTCGTTGAAGAGG |
| RT-qPCR | Mus musculus-*Ctsb*-F | AGACCTGCTTACTTGCTGTG |
| RT-qPCR | Mus musculus-*Ctsb*-R | GGAGGGATGGTGTATGGTAAG |
| RT-qPCR | Mus musculus-*Atg5*-F | ACCCCTGAAATGGCATTATCCAA |
| RT-qPCR | Mus musculus-*Atg5*-R | TGATGTTCCAAGGAAGAGCTGA |
| RT-qPCR | Mus musculus-*Atg7*-F | ACTTGACCGGTCTTACCCTG |
| RT-qPCR | Mus musculus-*Atg7*-R | TACTCCTGAGCTGTGGTTGC |
| RT-qPCR | Mus musculus-*Sqstm1*-F | CCGCCGCTTCAGCTTCTGCT |
| RT-qPCR | Mus musculus-*Sqstm1*-R | GTTCCCGCCGGCACTCCTTC |
| RT-qPCR | Mus musculus-*Map1lc3b*-F | ACAAAGAGTGGAAGATGTCCG |
| RT-qPCR | Mus musculus-*Map1lc3b*-R | CCCCTTGTATCGCTCTATAATCAC |
| RT-qPCR | Mus musculus-*Mitf*-F | AGGACCTTGAAAACCGACAG |
| RT-qPCR | Mus musculus-*Mitf*-R | GGTGGATGGGATAAGGGAAAG |
| RT-qPCR | Mus musculus-*Tfeb*-F | CTGTCCACTTCCAGTCGC |
| RT-qPCR | Mus musculus-*Tfeb*-R | CAGCAAACTTGTTCCCATAGG |
| RT-qPCR | Mus musculus-*Tfe3*-F | GCAGGCGATTCAACATTAACG |
| RT-qPCR | Mus musculus-*Tfe3*-R | GCTGTTCCTTCTGTAATTTGCG |
| RT-qPCR | Mus musculus-*Mcoln1*-F | TTGACAATAAAGCGCACAGTG |
| RT-qPCR | Mus musculus-*Mcoln1*-R | ACCACATCAAACAGAAGCCG |
| RT-qPCR | Mus musculus-*Ap1b1*-F | CGTTTGGATTTGTGGAGTCTG |
| RT-qPCR | Mus musculus-*Ap1b1*-R | TGTCACTGTTAAGCTCTGCC |
| RT-qPCR | Mus musculus-*Idua*-F | ATGCCCCAATCAACAGGAG |
| RT-qPCR | Mus musculus-*Idua*-R | GACATCCAGGTAAGTCACAGG |
| RT-qPCR | Mus musculus-*Arsa*-F | GCCCATCTGTATCTCTGTCATG |
| RT-qPCR | Mus musculus-*Arsa*-R | AGGTCATCCGCAAAGATCAG |
| RT-qPCR | Mus musculus-*Actb*-F | CTAAGGCCAACCGTGAAAAG |
| RT-qPCR | Mus musculus-*Actb*-R | ACCAGAGGCATACAGGGCA |
| RT-qPCR | Mus musculus-*Men1*-F | ACCTATCCATCATTGCTGCCCTCT |
| RT-qPCR | Mus musculus-*Men1*-R | ACCAGTTCGCGACTAGAAACACCT |
| RT-qPCR | Mus musculus-*Mll1*-F | TCTCAAACAGACTGACCAGC |
| RT-qPCR | Mus musculus-*Mll1*-R | CATCAGGAAACACAGCTCGT |
| RT-qPCR | Mus musculus-*mtAtp6*-F | GCCATTCCACTATGAGCTGGAGCC |
| RT-qPCR | Mus musculus-*mtAtp6*-R | GTGGAAGGAAGTGGGCAAGTGAGC |
| RT-qPCR | Mus musculus-*Tert*-F | CTAGCTCATGTGTCAAGACCCTCTT |
| RT-qPCR | Mus musculus-*Tert*-R | GCCAGCACGTTTCTCTCGTT |
| RT-qPCR | Mus musculus-*Tfam*-F | AAGGATGATTCGGCTCAGG |
| RT-qPCR | Mus musculus-*Tfam*-R | GGCTTTGAGACCTAACTGG |
| RT-qPCR | Mus musculus-*Ppargc1a*-F | CCCTGCCATTGTTAAGACC |
| RT-qPCR | Mus musculus-*Ppargc1a*-R | TGCTGCTGTTCCTGTTTTC |
| ChIP-qPCR | ChIP-*CTSE*-PP1-F | CCAGTTAGTAGAGCAATGGAGCA |
| ChIP-qPCR | ChIP-*CTSE*-PP1-R | CACAAACTGCACCCGTGTAAG |
| ChIP-qPCR | ChIP-*CTSE*-PP2-F | AGGGGTCAAAGGTCTAGGCT |
| ChIP-qPCR | ChIP-*CTSE*-PP2-R | TGGCAAGCAAACGTGGAGTA |
| ChIP-qPCR | ChIP-*CTSE*-PP3-F | GCCTTATCATTCGGCCCTCA |
| ChIP-qPCR | ChIP-*CTSE*-PP3-R | ATTGTGAGTCCGACCAGCAG |
| ChIP-qPCR | ChIP-*CTSB*-PP1-F | CCATCAAATAGCCATAACGCAT |
| ChIP-qPCR | ChIP-*CTSB*-PP1-R | ACACAGCCAGAACCAGGATAA |
| ChIP-qPCR | ChIP-*CTSB*-PP2-F | CAGAGTCCATGGCTGTCAGG |
| ChIP-qPCR | ChIP-*CTSB*-PP2-R | TCTGGAGCCCAAACATGGTC |
| ChIP-qPCR | ChIP-*CTSB*-PP3-F | TGCACTTTGTCCGTCCTCTC |
| ChIP-qPCR | ChIP-*CTSB*-PP3-R | GGAGCTGCGTCAGGATTTCT |
| ChIP-qPCR | ChIP-*CTSB*-PP4-F | TCCTGACGCAGCTCCTAG |
| ChIP-qPCR | ChIP-*CTSB*-PP4-R | AACAGCCTTTAGAAGGGCCA |
| ChIP-qPCR | ChIP-*CTSB*-PP5-F | GGACTTTTCTAGGCGGGGTG |
| ChIP-qPCR | ChIP-*CTSB*-PP5-R | CGAAACGCTGCGAATTGGG |
| ChIP-qPCR | ChIP-*MAP1LC3B*-PP1-F | CACACACCTGCTCCGCC |
| ChIP-qPCR | ChIP-*MAP1LC3B*-PP1-R | GCGATAGCCACTTCCCTTGT |
| ChIP-qPCR | ChIP-*MAP1LC3B*-PP2-F | ACTAGCGTAAGGTTGTCCAGC |
| ChIP-qPCR | ChIP-*MAP1LC3B*-PP2-R | CTACCGTTAGGTCGTCTGTGG |
| ChIP-qPCR | ChIP-*SQSTM1*-PP1-F | GTGAAGGCCTACCTTCTGGG |
| ChIP-qPCR | ChIP-*SQSTM1*-PP1-R | GTAGTGCGCCTGGAAGCC |
| ChIP-qPCR | ChIP-*SQSTM1*-PP2-F | ATGGCATGAGTTGGACCCAG |
| ChIP-qPCR | ChIP-*SQSTM1*-PP2-R | CAGAACTGGGGCAAGGAGAG |
| ChIP-qPCR | ChIP-*TFEB*-PP1-F | CCAGGCTGATCTGCTCCAAA |
| ChIP-qPCR | ChIP-*TFEB*-PP1-R | GGGTCTCCTCTCCCCATGAT |
| ChIP-qPCR | ChIP-*TFEB*-PP2-F | TTGTTGAGCAGAACAACGGC |
| ChIP-qPCR | ChIP-*TFEB*-PP2-R | TCTGTTGTTGCCCAAGTCGT |
| ChIP-qPCR | ChIP-*TFEB*-PP3-F | TGAGTGGGGCGTGAATGTAG |
| ChIP-qPCR | ChIP-*TFEB*-PP3-R | CCCCGATCATAAGGGGCAAG |
| ChIP-qPCR | ChIP-*TFEB*-PP4-F | AGCTGGAATTTGCCACAGGT |
| ChIP-qPCR | ChIP-*TFEB*-PP4-R | TGTCCGACCCCCTAAAAGGA |
| ChIP-qPCR | ChIP-*TFE3*-PP1-F | CCTAAGGAATCCGGCAGTGG |
| ChIP-qPCR | ChIP-*TFE3*-PP1-R | CTAGACGCAGTCAAGGGCTC |
| ChIP-qPCR | ChIP-*TFE3*-PP2-F | CTAGACGCAGTCAAGGGCTC |
| ChIP-qPCR | ChIP-*TFE3*-PP2-R | CTAGACGCAGTCAAGGGCTC |
| ChIP-qPCR | ChIP-*TFE3*-PP3-F | CTAGACGCAGTCAAGGGCTC |
| ChIP-qPCR | ChIP-*TFE3*-PP3-R | CTAGACGCAGTCAAGGGCTC |
| ChIP-qPCR | ChIP-*TFAM*-PP1-F | CCATGCCAGAATGGGAGGTT |
| ChIP-qPCR | ChIP-*TFAM*-PP1-R | TGGCTCTCTGGAAGTAGGACT |
| ChIP-qPCR | ChIP-*TFAM*-PP2-F | GGTCTCAAGACCACCGACAT |
| ChIP-qPCR | ChIP-*TFAM*-PP2-R | AGATTCCAAGGCCTTACCGC |
| shRNA | shNC | CCGGCAACAAGATGAAGAGCACCAACTCGAGTTGGTGCTCTTCATCTTGTTGTTTTT |
| shRNA | sh*MEN1*-1 | CCGGGCTGTACCTGAAAGGATCATACTCGAGTATGATCCTTTCAGGTACAGCTTTTTG |
| shRNA | sh*MEN1*-2 | CCGGCTGTACCTGAAAGGATCATACCTCGAGGTATGATCCTTTCAGGTACAGTTTTTG |
| siRNA | siNC | UUCUCCGAACGUGUCACGUTT |
| siRNA | si*MEN1*-1 | AAGGUCUCCGAUGUCAUAU |
| siRNA | si*MEN1*-2 | CUCUUCAGCUUCAUCACAGGC |
| siRNA | si*MLL1* | UGCCAAGCACUGUCGAAAUUA |
| siRNA | si*MLL4* | GCAUCAACUUCAAGCGGAAUU |
| siRNA | si*TFE3* | GCAGCUCCGAAUUCAGGAACU |
| siRNA | si*TFEB* | CUACAUCAAUCCUGAAAUG |
| siRNA | si*SETD2* | CCACAACUGAAGACUUUAATT |

**Supplementary Table S6**. Antibodies and Drugs.

| ITEM | REAGENT | SOURCE | IDENTIFIER | |
| --- | --- | --- | --- | --- |
| Antibody | Rabbit polyclonal anti-menin | Bethyl Laboratories | | A300-105A |
| Antibody | Mouse monoclonal anti-menin | Santa Cruz Biotechnology | | sc-374371 |
| Antibody | Rabbit monoclonal anti-CTSB | Cell Signaling Technology | | 31718 |
| Antibody | Rabbit polyclonal anti-CTSE | ABclonal Technology | | A2678 |
| Antibody | Mouse monoclonal anti-β-actin | Cell Signaling Technology | | 3700S |
| Antibody | Mouse monoclonal anti-LAMP1 | Cell Signaling Technology | | 15665 |
| Antibody | Rabbit monoclonal anti-TFE3 | ABclonal Technology | | A0548 |
| Antibody | Rabbit monoclonal anti-LC3B | abcam | | ab192890 |
| Antibody | Rabbit monoclonal anti-LC3A/B | Cell Signaling Technology | | 12741 |
| Antibody | Rabbit polyclonal anti-P62 | Proteintech | | 18420-1-AP |
| Antibody | Rabbit monoclonal anti-Phospho-Akt (Ser473) | Cell Signaling Technology | | 4060 |
| Antibody | Rabbit monoclonal anti-AKT | Cell Signaling Technology | | 9272S |
| Antibody | Rabbit monoclonal anti-Phospho-ERK1/2 (Thr202/Tyr204) | Cell Signaling Technology | | 4376 |
| Antibody | Rabbit polyclonal anti-ERK1/2 | Proteintech | | 16443-1-AP |
| Antibody | Rabbit polyclonal anti-Phospho-AMPK alpha 1/2 (Thr183/Thr172) | Zenbio | | 340763 |
| Antibody | Rabbit monoclonal anti-AMPK | Zenbio | | R23314 |
| Antibody | Rabbit monoclonal anti-Phospho-S6K (Thr389) | Cell Signaling Technology | | 9234 |
| Antibody | Rabbit polyclonal anti-S6K | Proteintech | | 14485-1-AP |
| Antibody | Rabbit monoclonal anti-GAPDH | ABclonal Technology | | A19056 |
| Antibody | Rabbit polyclonal anti-H3 | abcam | | ab1791 |
| Antibody | Rabbit monoclonal anti-H3K4me3 | Millipore | | 17-614 |
| Antibody | Rabbit monoclonal anti-H3K36me3 | Cell Signaling Technology | | 4909S |
| Antibody | Rabbit monoclonal anti-MLL1 | Cell Signaling Technology | | 14197 |
| Antibody | Rabbit polyclonal anti-MLL1 | Novus Biologicals | | NB600-256 |
| Antibody | Rabbit polyclonal anti-MLL4 | Bethyl Laboratories | | A300-113A |
| Antibody | Rabbit polyclonal anti-TFEB | Proteintech | | 13372-1-AP |
| Antibody | Mouse monoclonal anti-HA tag | abcam | | ab18181 |
| Antibody | Rabbit monoclonal anti-Flag tag | Cell Signaling Technology | | 14793 |
| Antibody | Mouse monoclonal anti-His | ABclonal Technology | | AE003 |
| Antibody | Rabbit monoclonal anti-LEDGF | Cell Signaling Technology | | 2088S |
| Antibody | Rabbit monoclonal anti-TFAM | ABclonal Technology | | A3173 |
| Antibody | Rabbit monoclonal anti-Tom20 | Cell Signaling Technology | | 42406 |
| Antibody | Rabbit monoclonal anti-COX IV | Cell Signaling Technology | | 4850 |
| Antibody | Mouse monoclonal anti-γH2A.X | Cell Signaling Technology | | 80312 |
| Antibody | Rabbit polyclonal anti-SPC | abcam | | ab40876 |
| Antibody for flow cytometry | 7-AAD | BD Biosciences | | 559925 |
| Antibody for flow cytometry | Anti-mouse CD31 PE-Cyanine7 | Biogems | | 03412-77 |
| Antibody for flow cytometry | CD45 Monoclonal Antibody (30-F11), PECyanine7 | eBioscience | | 25-0451-82 |
| Antibody for flow cytometry | CD326 (EpCAM) Monoclonal Antibody (G8.8), APC | eBioscience | | 17-5791-82 |
| Antibody for flow cytometry | MHC Class II (I-A/I-E) Monoclonal Antibody (M5 /114.15.2), eFluor 450 | eBioscience | | 48-5321-82 |
| Inhibitor | BafA1 | Selleck | | S1413 |
| Inhibitor | CQ | Sigma-Aldrich | | C6628 |
| Inhibitor | U0126 | Selleck | | S1102 |
| Inhibitor | LY294002 | Sigma-Aldrich | | 440202 |
| Inhibitor | SP2509 | Selleck | | S7680 |
| Inhibitor | Revumenib | MedChemExpress | | HY-136175 |
| Inhibitor | MI-3 | Selleck | | S7619 |
| Inhibitor | Sinefungin | MedChemExpress | | HY-101938 |
| Inhibitor | JIB-04 | MedChemExpress | | HY-13953 |
| Inhibitor | EZM0414 | MedChemExpress | | HY-136328 |
| Inhibitor | CCCP | Selleck | | S6494 |
| Drug | G418 | Gibco | | 10131035 |
| Drug | Puro | Gibco | | A1113803 |
