# Supplementary material for: Menin maintains lysosomal and mitochondrial homeostasis through epigenetic mechanisms in lung cancer
Source: Cell Death Dis. 2025 Mar 8;16(1):163. doi: 10.1038/s41419-025-07489-0 (PMC11890858; doi:10.1038/s41419-025-07489-0)
Supplement: Supplementary file 3 — Supplementary Materials and Methods [file 41419_2025_7489_MOESM3_ESM.docx]

**Supplementary Materials and Methods**

**Cell culture**

Cells were cultured at 37℃ with 5% CO_2_, following sterile techniques. All cell lines were authenticated by STR profiling within 6 months and routinely tested for Mycoplasma infection using a Myco-Lumi Luminescent Mycoplasma Detection Kit (Beyotime, C0297S). All culture media contained 10% FBS (VivaCell, C04001), 100 U/ml penicillin-streptomycin (Gibco, 10378016), and 2 mM glutamine (Gibco, A2916801). A549 was cultured in F12-K medium (BasalMedia, L450KJ), while NCI-H157, 16-HBE, NCI-H1975, THP-1 and MV4-11 were cultured in 1640 medium (BasalMedia, L210KJ). MEFs, GP2-293, HEK-293T, and A375 were cultured in DMEM medium (BasalMedia, L110KJ). HepG2 and SK-Hep-1 were cultured in MEM medium (BasalMedia, L550KJ). A549, NCI-H1975, SK-Hep1, THP-1, A375 and HEK-293T cells were obtained from National Collection of Authenticated Cell Cultures. NCI-H157, 16-HBE, MV4-11, GP2-293 and HepG2 were obtained from ATCC. The Eagle's Balanced Salt Solution (EBSS) (BasalMedia, B610KJ) supplemented with serum and glucose was used for AA-star. The glucose-free DMEM (BasalMedia, L160KJ) supplemented with serum was used for glu-star.

**Isolation of primary cells**

MEFs were isolated using previously established methods [1]. After a 2-day treatment with 4-OH TAM (Sigma-Aldrich, H6278), cellular DNA was extracted to verify the genotype using the genotyping PCR primers (Listed in Supplementary Table S5). ATII cells were isolated from mice with the desired genotype that were hybridized with the *Sftpc-Cre* system. The general procedure involved euthanizing the mice 10 days after TAM injection, preparing lung cell suspensions, and utilizing anti-MHCII and anti-EpCAM antibodies (Listed in Supplementary Table S6) for flow cytometry sorting of ATII cells [2].

**Construction of shRNA plasmids**

The plasmid backbone was pLKO.1 (Addgene, 8453; Bob Weinberg). The shNC served as the negative-control sequence for gene-knockdown experiments mediated by shRNA. The sequences are listed in the Supplementary Table S5. The plasmids containing the shRNA sequences, along with packaging plasmids psPAX2 (Addgene, 12260; Didier Trono) and pMD2.G (Addgene, 12259; Didier Trono), were co-transfected into HEK-293T cells. After 5 h, the medium was replaced with free complete culture medium, and the viral supernatant was collected after 48 h.

**Construction of expression plasmids**

The plasmid backbone was pLNCX2 (Takara bio, 631503) [1]. The PCR primers for cloning the target gene are listed in the Supplementary Table S5. The plasmids, along with pVSVG (Addgene, 138479; Akitsu Hotta), were co-transfected into GP2-293 cells. The collected viral supernatant was filtered and used to infect the corresponding cells.

**Gene transfection**

The lipofectamine transfection reagent (Yeasen, 40802ES03) was used for the siRNA and plasmid transfection according to the manufacturer's instructions. The siNC served as the negative-control sequence for gene-knockdown experiments mediated by siRNA. All siRNA sequences (GenePharma) for the respective genes are listed in the Supplementary Table S5.

**Western blotting**

Western blotting experiments were performed according to our previous report [3]. Briefly, the cells were lysed using RIPA lysis buffer (Solarbio, R0020) supplemented with 1 mM PMSF (Solarbio, P0100) and phosphatase inhibitor (Roche, 4906837001) to get total proteins. Nuclear and cytoplasmic proteins were extracted by the kit (Beyotime, P0027). The denatured proteins were separated by SDS-PAGE and transferred onto PVDF transfer membranes (Millipore, ISEQ00010). The membranes were then incubated with specific antibodies (Listed in Supplementary Table S6; 1:500-1:1000­). After washing, the membranes were incubated with a secondary antibody (Invitrogen, 31460 or 32430; 1:5000). Immunoreactive bands were visualized using an ECL detection kit (Millipore, WBULP) following the manufacturer's instructions. Protein marker (ABclonal Technology, RM02949) was used to visualize the band range of the target protein. All immunoblotting experiments were performed at least three times to ensure reproducibility.

**Immunohistochemistry (IHC)**

IHC and hematoxylin-eosin (HE) experiments were performed according to our previous report [1]. Three groups of mouse models were involved in these experiments. Group 1 consisted of WT (n = 6, m:f = 3:3), MS (n = 6, m:f = 3:3), KS (n = 8, m:f = 4:4), KMS (n = 8, m:f = 4:4). Group 2 consisted of WT (n = 9, m:f = 4:5) and *Men1^∆/∆^* (n = 9, m:f = 5:4). Group 3 consisted of solvent control KMS (n = 8, m:f = 4:4) and SP2509-treated KMS (n = 14, m:f = 6:8). For the IHC, mouse lung tissues were fixed with 4% paraformaldehyde, dehydrated, embedded in paraffin, and sectioned into 4 μm thick slices. Antigen retrieval was performed using high-pressure boiling for 10 min. The samples were incubated with the antibodies at 4℃ for 12 h. Detailed information about the antibodies is listed in Supplementary Table S6. Finally, the sections were photographed, and ImageJ software was used for quantification and analysis of the positive area.

**Immunofluorescence (IF)**

IF assays were performed following the protocol described in our previous report [1]. Briefly, the cells were seeded on slides (NEST, 801011) one day prior. For nuclear proteins, cells were fixed with 4% paraformaldehyde for 10 min at room temperature. For cytoplasmic proteins, cells were fixed with 100% methanol at -20℃ for 15 min. PBS containing 5% goat serum and 0.3% Triton-X100 served as the Blocking solution. Samples were incubated with the antibodies (Listed in Supplementary Table S6; 1:100) at 4℃ for 12 h. Secondary antibodies (Abcam, ab150116, ab6717, ab175652) were applied at a dilution of 1:200. Mounting medium with DAPI (VECTASHIELD, H-1200) was used to visualize the nucleus. Images were captured using a fluorescence microscope.

**Real-time quantitative mRNA detection (RT-qPCR)**

RNA was isolated with Total RNA Extraction Reagent (Yeasen, 10606ES60) and reverse transcribed into cDNA using HiScript III RT SuperMix for qPCR (Vazyme, R323) according to﻿ manufacturer’s protocol. Transcript quantification was performed by RT-qPCR using SYBR green master mix (Vazyme, Q712) in the real-time PCR system. Primer sequences are available in Supplementary Table S5. Each RT-qPCR experiment was conducted with three biological replicates.

**RNA-Seq analysis**

Control and *MEN1*-KD A549 cells were collected in Total RNA Extraction Reagent with three biological replicates per group. The samples were then outsourced to Novogene (Beijing, China) for RNA-Seq and subsequent data analysis. Briefly, RNA samples underwent strict quality control primarily through the Agilent 2100 bioanalyzer prior to library preparation. Eukaryotic poly(A) mRNA was selected for library construction. Illumina sequencing was carried out on the NovaSeq 6000 platform. Sequencing reads were transformed from the image data obtained by the high-throughput sequencer into sequence data (reads) by CASAVA base calling, with files in fastq format containing the sequence information of the sequencing reads and their corresponding sequencing quality information. Differentially expressed genes were defined as those with an adjusted P-value < 0.01 and a fold-change < 2/3 or >3/2. Gene Set Enrichment Analysis (GSEA) was performed using GSEA (4.0.3) software to identify the association of *MEN1* expression with the autophagy and mitochondria gene signature.**﻿**

**Co-immunoprecipitation (Co-IP) assays**

Co-IP assays were performed using the Flag-tag Protein IP Assay Kit (Beyotime, P2181S) or HA-tag Protein IP Assay Kit (Beyotime, P2185S) following the manufacturer's instructions [1]. Briefly, cells were lysed in a lysis buffer supplemented with the protease inhibitor cocktail. The lysates were then centrifuged, and 1 mg of protein lysate was incubated with Anti-Flag Magnetic Beads or Anti-HA Magnetic Beads, depending on the tag used. The lysates were immunoprecipitated with the respective beads at 4℃ for 12 h. After incubation, the beads were washed three times with the lysis buffer. The bound proteins were eluted using sodium dodecyl sulfate sample buffer and subjected to western blotting. The antibodies used for the Co-IP assays are listed in Supplementary Table S6.

**﻿Protein quantification**

Soluble and insoluble protein extraction was performed as previously reported [4]. One million cells of each experimental group were lysed in lysis buffer (50 mM TRIS-HCl, pH 7.4, 0.1% NP-40, 1 mM EDTA, 1% glycerol) containing 1 mM PMSF. After 30 min of ice-cold lysis and centrifugation (12,000 g, 10 min, 4℃), the supernatant was collected as the soluble protein fraction, and the pellet was collected as the insoluble protein fraction. The pellet was further lysed in RIPA lysis buffer with PMSF and sonicated for 30 s at 20% power. The protein concentration was measured using the BCA assay kit (Thermo Scientific, 23227). Three biological replicates were carried out for each experimental group. Finally, both fractions were mixed with Loading Buffer (Beyotime, P0015F), boiled for 10 min, and used for gel electrophoresis. Coomassie Brilliant Blue staining was performed to analyze protein deposition in the cells.

**PAS and﻿** **Oil Red O staining**

PAS and﻿ Oil Red O staining was performed according to the previous report [5]. For PAS staining, cells were fixed with 70% ethanol for 10 min and paraffin sections of lung tissue required dewaxing and rehydration before staining. PAS staining was performed using the Periodic Acid-Schiff Staining Kit (Beyotime, C0142S). For Oil Red O staining, cells or frozen sections of lung tissue were fixed with 4% paraformaldehyde for 10 min. The experiments were carried out using the Modified Oil Red O Staining Kit (Beyotime, C0158S) according to the manufacturer's protocol. A group of mouse models was involved in these experiments: WT (n = 6, m:f = 3:3), MS (n = 6, m:f = 3:3), KS (n = 8, m:f = 4:4), KMS (n = 8, m:f = 4:4). Photographs of the samples were taken, and the positive area was quantitatively analyzed using Image J software.

**Public database analysis**

The transcriptomic raw data of TCGA-LUAD were downloaded from The Cancer Genome Atlas (TCGA) website. DESeq2 and clusterProfiler packages in R (4.4.1) software were used for KEGG analysis. ChIP-seq data were obtained from the GEO database (GSE194353). Gene visualization was performed using the Integrative Genomics Viewer (2.19.1).

**Reference**

1. Qiu H, Jin BM, Wang ZF, Xu B, Zheng QF, Zhang L*, et al.* MEN1 deficiency leads to neuroendocrine differentiation of lung cancer and disrupts the DNA damage response. Nat Commun. 2020; 11**:**1009.

2. Hasegawa K, Sato A, Tanimura K, Uemasu K, Hamakawa Y, Fuseya Y*, et al.* Fraction of MHCII and EpCAM expression characterizes distal lung epithelial cells for alveolar type 2 cell isolation. Respir Res. 2017; 18**:**150.

3. Xu B, Li SH, Zheng R, Gao SB, Ding LH, Yin ZY*, et al.* Menin promotes hepatocellular carcinogenesis and epigenetically up-regulates Yap1 transcription. Proc Natl Acad Sci U S A. 2013; 110**:**17480-17485.

4. Shrestha A, Brunette S, Stanford WL, Megeney LA. The metacaspase Yca1 maintains proteostasis through multiple interactions with the ubiquitin system. Cell Discov. 2019; 5**:**6.

5. Scur M, Mahmoud AB, Dey S, Abdalbarri F, Stylianides I, Medina-Luna D*, et al.* Alveolar macrophage metabolic programming via a C-type lectin receptor protects against lipo-toxicity and cell death. Nat Commun. 2022; 13**:**7272.
