# Supplementary material for: Menin maintains lysosomal and mitochondrial homeostasis through epigenetic mechanisms in lung cancer
Source: Cell Death Dis. 2025 Mar 8;16(1):163. doi: 10.1038/s41419-025-07489-0 (PMC11890858; doi:10.1038/s41419-025-07489-0)

Original western blotting bands

All exposure images of western blotting were obtained through film.

Figure 1F

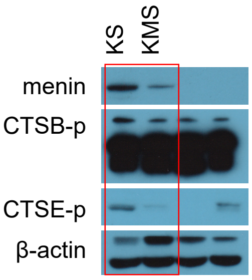

Figure 2C

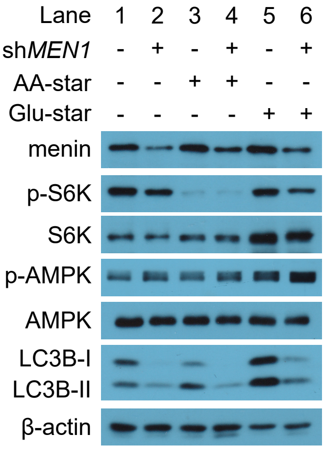

Figure 2F

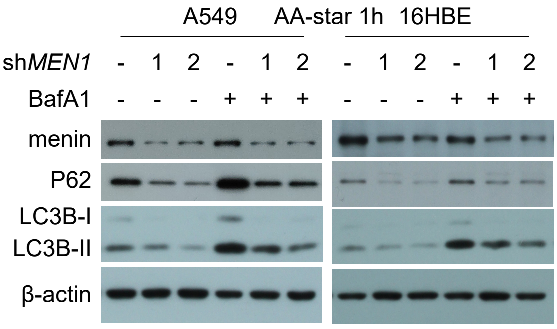

Figure 2G

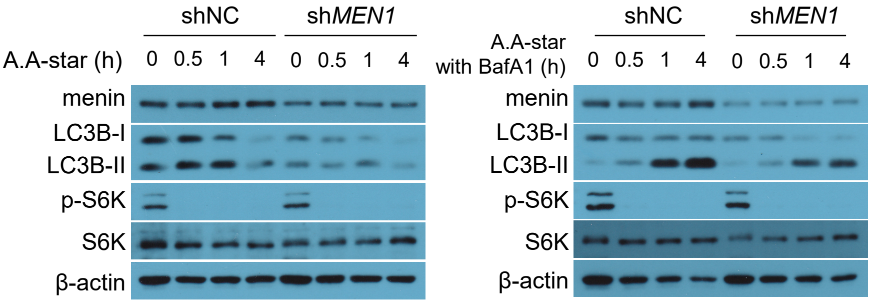

Figure 4A

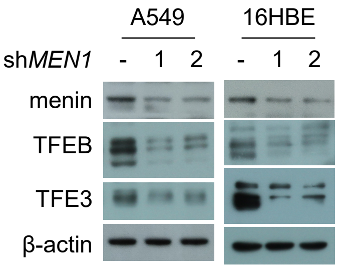

Figure 4B

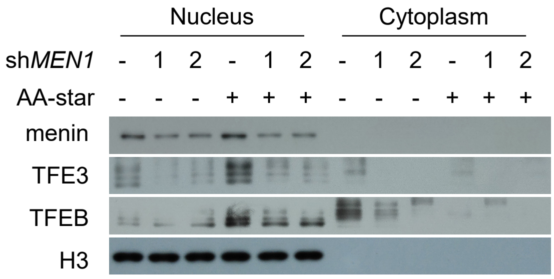

Figure 4F

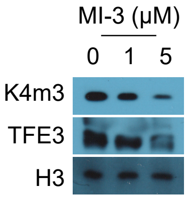

Figure 4G

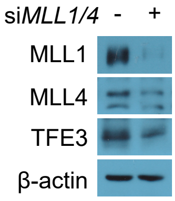

Figure 4J

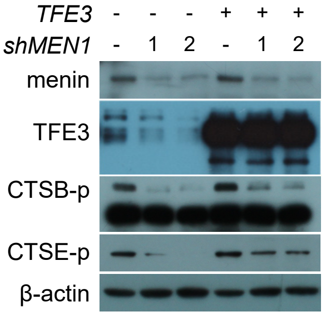

Figure 5M

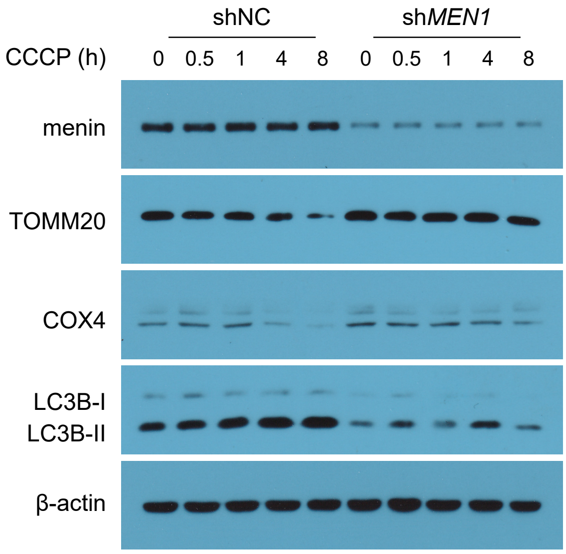

shNC
sh*MEN1*
CCCP (h)
0 0.5 1 4 8 0 0.5 1 4 8
menin
TOMM20
COX4
LC3B-I
LC3B-II
β-actin

Supplementary figure S1B

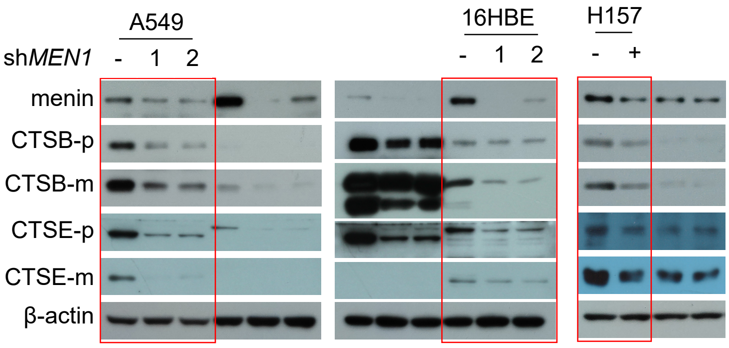

Supplementary figure S1J

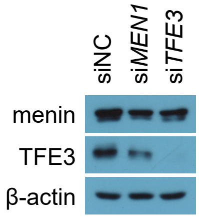

Supplementary figure S2C

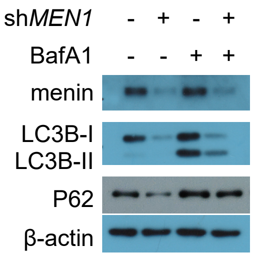

Supplementary figure S2D

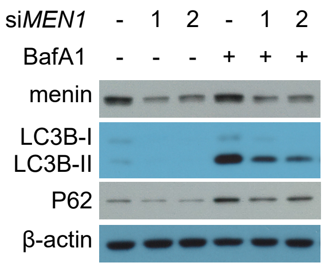

Supplementary figure S2E

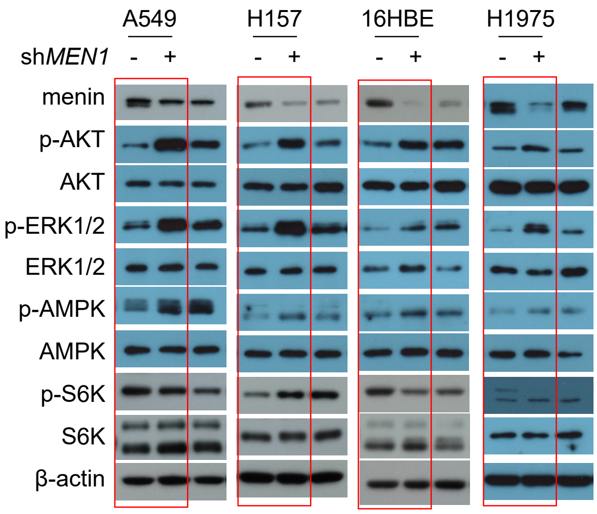

Supplementary figure S2F

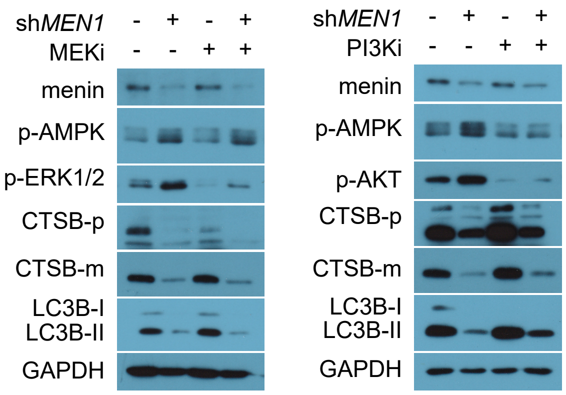

Supplementary figure S2G

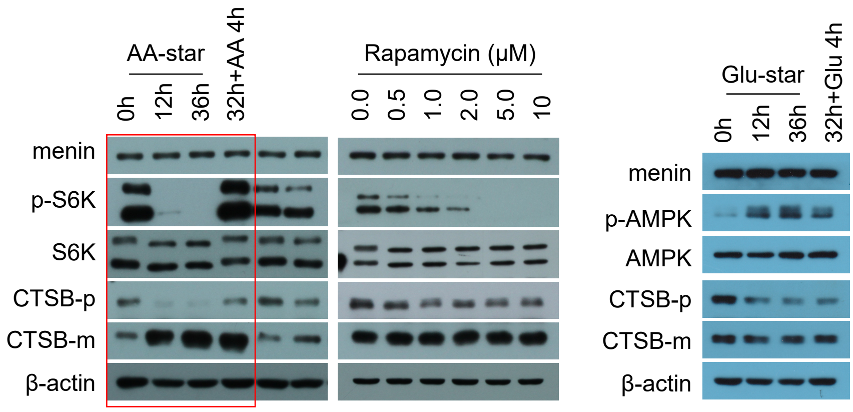

Supplementary figure S3A

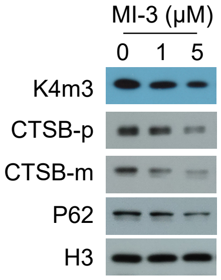

Supplementary figure S3B

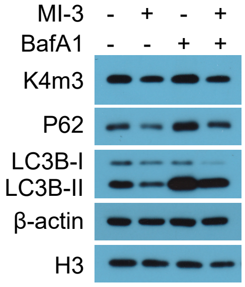

Supplementary figure S3C

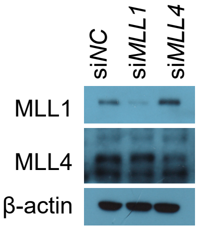

Supplementary figure S3D

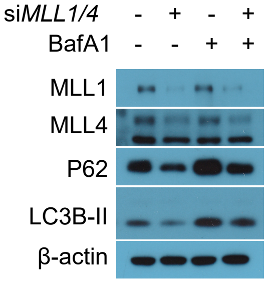

Supplementary figure S3I

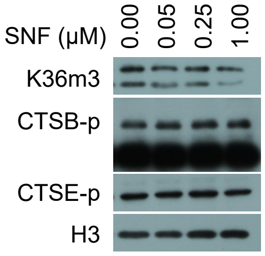

Supplementary figure S3K

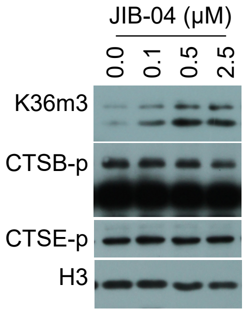

Supplementary figure S3M

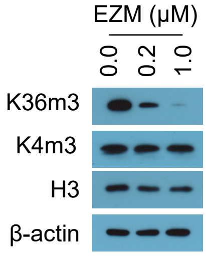

Supplementary figure S4A

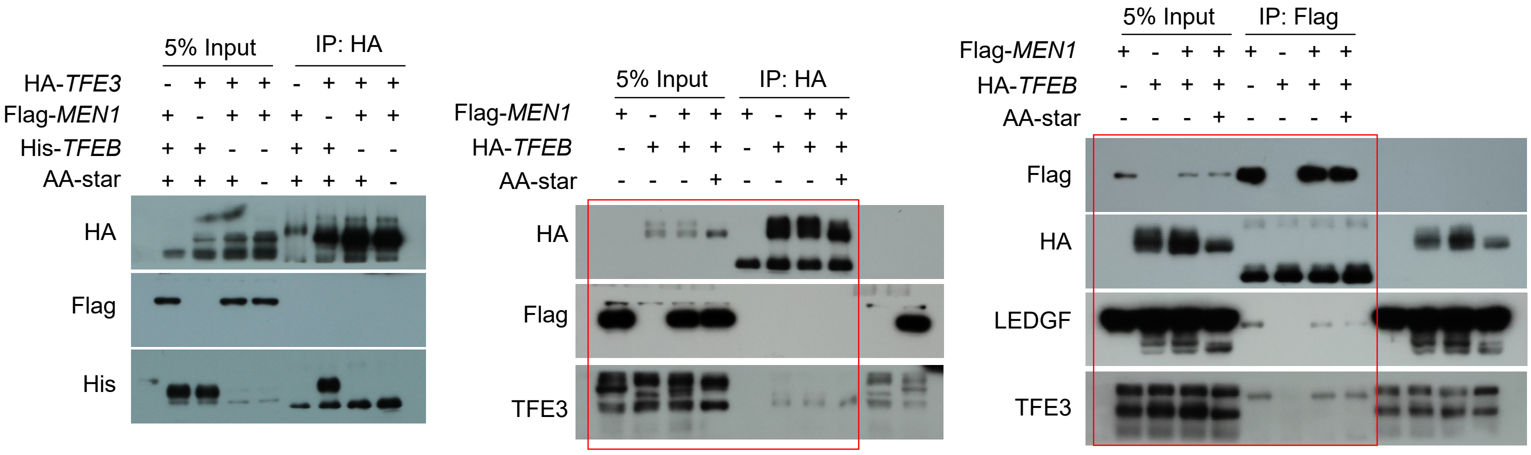

Supplementary figure S5B

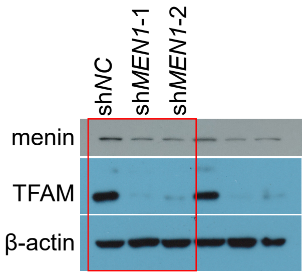

Supplementary figure S5C

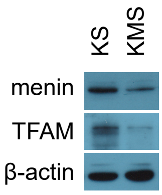

Supplementary figure S5D

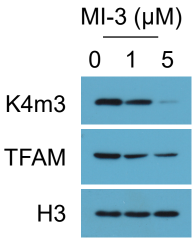

Supplementary figure S6H

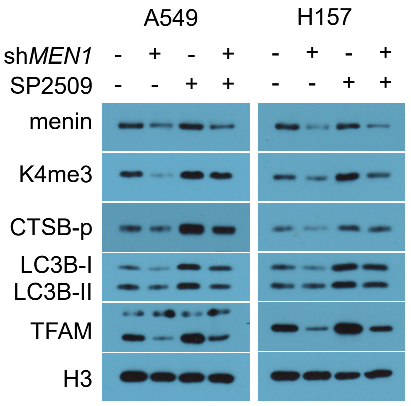

Supplementary figure S6I

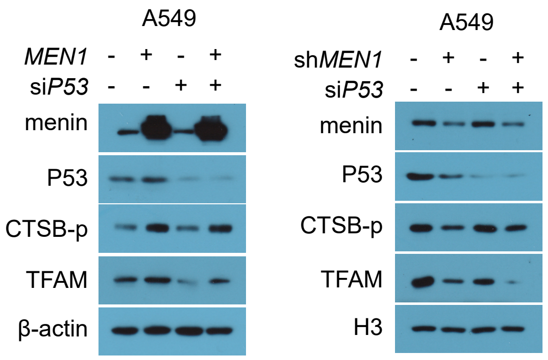

Supplement: Supplementary file 4 — Supplemental Material-Original western blotting bands [file 41419_2025_7489_MOESM4_ESM.pdf]
